# Host gut resistome in Gulf War chronic multisymptom illness correlates with persistent inflammation

**DOI:** 10.1038/s42003-022-03494-7

**Published:** 2022-06-07

**Authors:** Dipro Bose, Somdatta Chatterjee, Ethan Older, Ratanesh Seth, Patricia Janulewicz, Punnag Saha, Ayan Mondal, Jeffrey M. Carlson, Alan W. Decho, Kimberly Sullivan, Nancy Klimas, Stephen Lasley, Jie Li, Saurabh Chatterjee

**Affiliations:** 1 Environmental Health and Disease Laboratory, Department of Environmental Health Sciences, Arnold School of Public Health, University of South Carolina, Columbia, SC USA; 2 Department of Chemistry and Biochemistry, University of South Carolina, Columbia, SC USA; 3 Department of Environmental Health, Boston University School of Public Health, Boston, MA USA; 4 Department of Clinical Immunology, Nova Southeastern University, Fort Lauderdale, FL USA; 5 Department of Cancer Biology and Pharmacology, University of Illinois College of Medicine, Peoria, IL USA; 6 Columbia VA Medical Center, Columbia, SC USA

**Keywords:** Translational research, Microbiota

## Abstract

Chronic multisymptom illness (CMI) affects a subsection of elderly and war Veterans and is associated with systemic inflammation. Here, using a mouse model of CMI and a group of Gulf War (GW) Veterans’ with CMI we show the presence of an altered host resistome. Results show that antibiotic resistance genes (ARGs) are significantly altered in the CMI group in both mice and GW Veterans when compared to control. Fecal samples from GW Veterans with persistent CMI show a significant increase of resistance to a wide class of antibiotics and exhibited an array of mobile genetic elements (MGEs) distinct from normal healthy controls. The altered resistome and gene signature is correlated with mouse serum IL-6 levels. Altered resistome in mice also is correlated strongly with intestinal inflammation, decreased synaptic plasticity, reversible with fecal microbiota transplant (FMT). The results reported might help in understanding the risks to treating hospital acquired infections in this population.

## Abstract (web summary)

Analysis of fecal samples from patients and mice, shows that chronic multisymptom illness which affects a subsection of elderly and war Veterans, is associated with alteration in genes related to antibiotic resistance.

## Introduction

Antibiotic resistance has emerged as a threat to public health on the local and global scale^1,2^. Resistance to antibiotics is conferred on bacteria by ARGs. All of the ARGs collectively form a resistome, and usually are carried by the opportunistic pathogens humans may encounter, thus increasing the risk of acquired infections that are difficult to treat with currently approved antibiotics^3–6^. The evolution of drug resistance in such pathogens is driven by chromosomal mutation and the acquisition of ARGs. Since most of the ARGs are linked to MGEs, they can transfer easily through horizontal gene transfer (HGT) among different clones, taxa and habitats^7–10^.

The human gut harbors multiple commensal microorganisms and forms a good reservoir of ARGs. The gut environment predisposes ARG transfer, sometimes leading to emergence and spread of specific bacterial clones carrying genes of resistance and/or virulence. The most frequently reported genes are those directed against tetracycline, β-lactams, aminoglycosides, and glycopeptides. Tetracycline and glycopeptide resistant genes are most common in fecal samples of human^4,11–13^. On the other hand, human microbiota is also influenced by environmental factors like exposure to chemicals (pesticide, biocide) along with antibiotics that can alter the microbial composition and affect colonization resistance to pathogens. The selection pressure due to the exposure to such chemicals have resulted in emergence and increase of ARGs in both environment as well as gut microbiome^14–18^. A report from Sun et al., shows that changes in living environment can alter the human gut microbiota and resistome^12^. Another similar study by Gao et al. suggested that exposure to Triclosan resulted in gut microbiome and resistome alteration that persisted over a prolonged exposure^19,20^.

Numerous studies have reported that elderly and war Veterans often suffer from multiple syndromes with inflammatory phenotypes and are more susceptible to resistance against a large number of antibiotics^21–23^. Veterans have been reported to be resistant to amoxicillin, β-lactams, fluoroquinolones, methicillin and most importantly the carbapenem group of antibiotics^24,25^. In our present study, we aimed to analyze the ARG and MGE patterns in a Gulf War Illness (GWI) mouse model as well as in a cohort of GW Veterans. GWI is a complex multisymptom illness often closely associated with chronic multisymptom illnesses but now classified exclusively as GWI. The above referred condition are reported by a section Veterans who returned from Operation Desert Shield/Desert Storm in 1990–1991. Symptoms reported by the GW Veterans include fatigue, headache, cognitive dysfunction, musculoskeletal pain, respiratory and gastrointestinal dysfunctions more often characterized by a persistent systemic inflammation with higher IL-6, TNF-R1 and IL-1β blood levels. These symptoms have been linked to chemical exposures experienced during the war^26–28^.

Several studies in preclinical mouse models of GWI have reported that exposure to chemicals such as insecticides and anti-nerve gas agents resulted in gut microbial dysbiosis. There has been a decrease in the relative abundance of several beneficial bacteria. Recently, a study in GWI Veterans also reported similar alteration of gut microbiome^29–32^. With reported studies on microbial dysbiosis patterns in the preclinical animal models as well as the GW Veterans well established, and with studies suggesting the potential of environmental chemicals in increasing antibacterial resistance, we hypothesized that exposure to environmental pesticides and pyridostigmine bromide (PB) may also lead to an alteration of ARGs and MGEs expression, an important constituent of the gut resistome. In the present study we used both GW Veteran stool samples and fecal pellets from a GWI persistence mouse model administered with representative GW chemicals to study the alteration in gut resistome. We also studied the possible associations between gut resistome and GWI proinflammatory pathology by using a prolonged fecal microbiota transfer regimen that aimed to recolonize the mouse gut with microbiota from a healthy donor.

## Results

### Characterization of antimicrobial resistance in mouse fecal samples

**Fig. 1:**
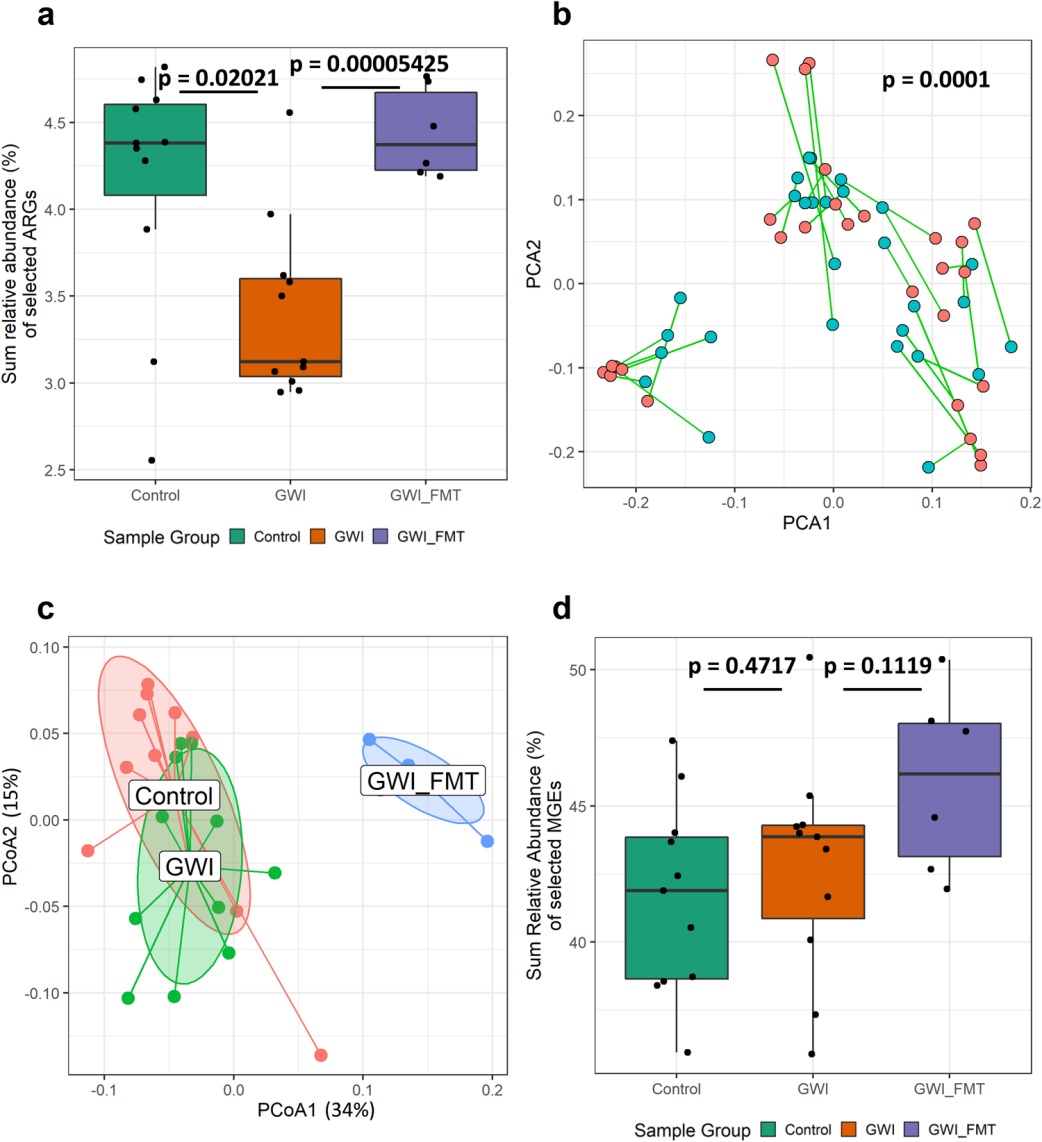
Distribution of ARGs and MGEs in persistence GWI mouse models. **a** Box plots showing sum relative abundance (%) of selected antibiotic resistance genes (ARGs) in Control (adult C57BL/6 J mice administered with vehicle, *n* = 11 biologically independent samples), GWI (adult C57BL/6 J mice administered with GW chemicals, *n* = 11 biologically independent samples) and GWI_FMT (adult C57BL/6 J mice group exposed to GW chemicals followed by Fecal Microbiota Transfer (FMT) treatment, *n* = 6 biologically independent samples). Median values of 4.38% in Control, 3.12% in GWI, and 4.37% in GWI_FMT indicated by solid black lines. **b** Procrustes rotation analysis comparing resistome (blue) and microbiome (red) changes using PCA ordinations, (*X*-axis: PCA1, *Y*-axis: PCA2, PROTEST, *M*^2^ = 0.4302, *p* = 0.0001). **c** PCoA of Bray-Curtis dissimilarity between ARG and Taxa profiles across sample groups. Principal coordinate axes PCoA1 (34%) and PCoA2 (15%) are calculated to explain 49% of variance detected. Lines connect points from the center of gravity of each sample group. **d** Box plots showing sum relative abundance (%) of selected mobile genetic elements (MGEs) Control, GWI and GWI_FMT. Boxes in the box plots indicate interquartile range. Error bars in the box plots extend to the most extreme values within 1.5 times the interquartile range. Outlier data points are represented by black dots drawn outside of the box plot. *p*-values were determined using PROTEST or Welch’s *t*-test where *p* < 0.05 was considered statistically significant.

We performed whole-genome shotgun sequencing, metagenomic assembly, and functional gene annotation on fecal samples collected from three groups (Control, GWI, and GWI_FMT) to construct ARG profiles associated with the GWI mice model and the FMT treatment. We detected 455 unique ARGs across all groups with 538 ± 16 (mean ± standard error) total ARGs in Control, 514 ± 15 total ARGs in GWI, and 567 ± 19 total ARGs in GWI_FMT. Performing PERMANOVA revealed a significant deviation in the ARG profiles across sample groups (*p* = 0.0001, *R*^2^ = 34%). This indicated that the resistome profile of each sample group were distinct from each other. To get a clearer picture of the specific changes in the resistome, we performed differential abundance analysis of the ARGs to identify significantly changed genes (DESeq2, negative binomial generalized linear models (GLMs), Wald’s test, *p* < 0.05). Comparing these selected ARGs between sample groups, we observed a significant decrease in their sum relative abundances from Control to GWI (*p* = 0.02021, Welch two-sample *t*-test) and a significant increase when comparing GWI to GWI_FMT group (*p* = 0.00005425, Welch two-sample *t*-test, Fig. 1a).

Looking to connect ARG changes with gut microbiome composition, we performed procrustes rotation of the principal component analyses (PCAs) of the resistome and microbiome profiles which showed a significant correlation (*p* = 0.0001, PROTEST, *M*^2^ = 0.4302, Fig. 1b), confirming that resistome changes were in fact linked to microbial dysbiosis. Further, principal coordinate analysis of ARG profiles showed clustering of profiles according to sample group with the first two axes accounting for 49% of the variance in the ARG profiles. The GWI_FMT group formed an independent cluster from both GWI and Control groups (ape, pcoa, PCoA1 accounting for 34% of variance in ARG profile, Fig. 1c).

We also examined the profiles of MGEs constituting the mobilome in each sample group. We detected 77 unique MGEs evenly distributed across all groups with 77 ± 3 total MGEs in Control, 74 ± 2 total MGEs in GWI, and 81 ± 3 total MGEs in GWI_FMT. PERMANOVA showed significant differences in the mobilomes with respect to sample group, supporting the observed deviations in resistome profile which we previously linked to changes in the microbiome composition (*p* = 0.0001, *R*^2^ = 24%). We selected differentially abundant MGEs (DESeq2, negative binomial GLMs, Wald’s test, *p* < 0.05), compared their sum relative abundances as previously described for ARGs. The changes observed in the sum relative abundances between Control to GWI (*p* = 0.4717, Welch two-sample *t*-test) and between GWI and GWI_FMT (*p* = 0.1119, Welch two-sample *t*-test, Fig. 1d) were not statistically significant. Hence, on administration of representative GW chemicals in GWI mice model, we did not see an increase in the relative abundance of ARGs and the changes observed in the relative abundances of MGEs were not significant.

### Distribution of ARGs and MGEs across the different groups in GWI mouse model

**Fig. 2:**
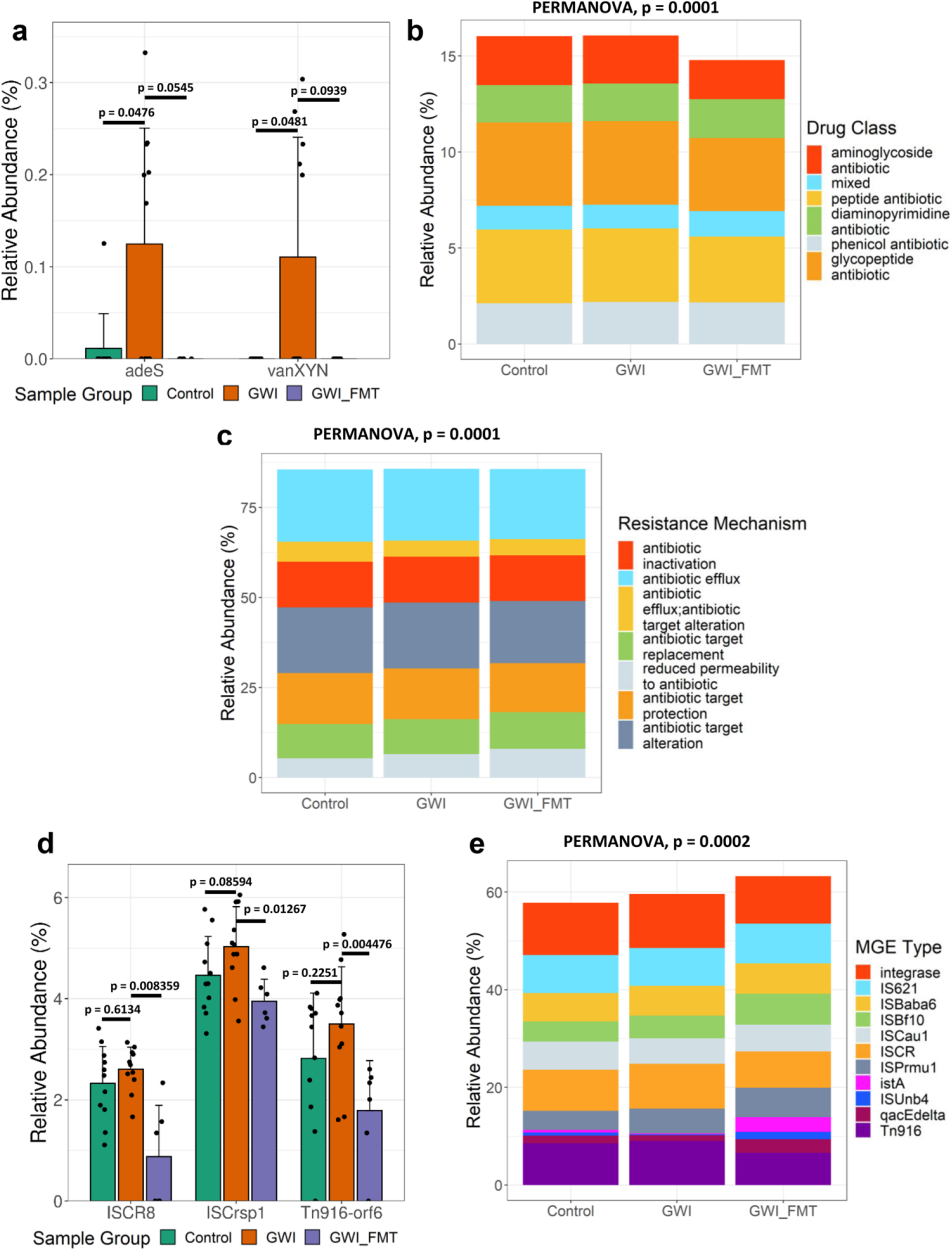
Classification of selected ARGs and MGEs in persistence GWI mouse model. **a** Grouped bar graph showing relative abundance (%) of selected ARGs in Control (adult C57BL/6 J mice administered with vehicle, *n* = 11 biologically independent samples), GWI (adult C57BL/6 J mice administered with GW chemicals, *n* = 11 biologically independent samples), and GWI_FMT (adult C57BL/6 J mice group exposed to GW chemicals followed by FMT treatment, *n* = 6 biologically independent samples) groups. Stacked bar analysis of relative abundance (%) of **b** drug classes resistances and **c** mechanisms of resistance. **d** Grouped bar graph showing relative abundance (%) of selected MGEs in Control, GWI and GWI_FMT groups. **e** Stacked bar analysis of relative abundance (%) of MGE types. Data represented as Mean±SD (SD: Standard deviation) for the bar graphs. *p*-values were determined using Wald’s test or PERMANOVA where *p* < 0.05 was considered statistically significant.

We detected multiple ARGs imparting resistance to antimicrobial classes which have been marked as highly important and critically important by the World Health Organization (WHO) (AGISAR, 2018). In our analysis, glycopeptide resistance genes were the most abundant ARG observed across all three mice groups (not statistically significant). Using differential abundance analysis, we identified the individual ARGs which were significantly altered (DESeq2, negative binomial GLMs, Wald’s test, *p* < 0.05). Among these ARGs*, vanXYN (*ARO: 3002969), and *adeS* (ARO: 3000549) were significantly increased in the GWI group compared to the Control group (DESeq2, negative binomial GLMs, Wald’s test, *p* < 0.05, Fig. 2a). This result is suggestive of a potential effect of GW chemicals on the individual ARGs. FMT treatment decreased the abundance of *vanXYN* and *adeS* genes as observed in the GWI_FMT group over the GWI group, however it was not statistically significant. Interestingly, there were 6 unique ARGs which were only present in the mouse GWI group.

When comparing the resistant drug classes across the three groups, it was observed that glycopeptide antibiotics followed by aminoglycosides, peptide, phenicol, and diaminopyrimidine antibiotics were among the resistant drug classes that were detected in the 3 experimental mice groups (*p* = 0.0001, PERMANOVA, *R*^2^ = 33%, Fig. 2b). Based on their mechanisms of resistance, antibiotic efflux, antibiotic target alteration, antibiotic target protection, and antibiotic inactivation mechanisms were among the antibiotic resistance mechanisms that were detected in the 3 experimental mice groups (*p* = 0.0001, PERMANOVA, *R*^2^ = 31%, Fig. 2c).

ARGs have a high propensity to mobilize from one bacterium to another through the MGEs by horizontal gene transfer^9^. We thus investigated the presence of different MGEs and MGE types among the three mice groups. We observed that relative abundances of *ISCR8, ISCrsp1,* and *Tn916-orf6* increased (not statistically significant) in the GWI group compared to Control but significantly decrease in the GWI_FMT group (DESeq2, negative binomial GLMs, Wald’s test, *p* < 0.05, Fig. 2d). Transposase, insertional sequences *ISCR* and integrase were among the abundant MGE types detected in all the samples across the 3 mice groups (Fig. 2e). Multivariate analysis of the MGE type profiles showed a significant deviation and correlation pattern that was dependent on sample groups (*p* = 0.0002, PERMANOVA, *R*^2^ = 33%).

### Demographic Information of GW Veterans

In this study, we obtained samples from 33 GW Veterans. Out of these 33 participants, 28 met the required Kansas GWI Criteria and were categorized into the Hum_GWI group. The remaining 5 participants did not meet the criteria and were considered as the Hum_Control group. There were no major differences based on age, height, weight, or body mass index of the participants in both groups (Supplementary Table 2). In the Hum_GWI group, 61% of the participants reported diarrhea and nausea, 57% reported abdominal pain or cramping, 46% reported irritable bowel syndrome (IBS). In the Hum_Control group, there were no reports of diarrhea or IBS, but 40% of participants reported nausea and 20% reported abdominal pain or cramping.

### Characterization of antimicrobial resistance gene in Veteran samples

**Fig. 3:**
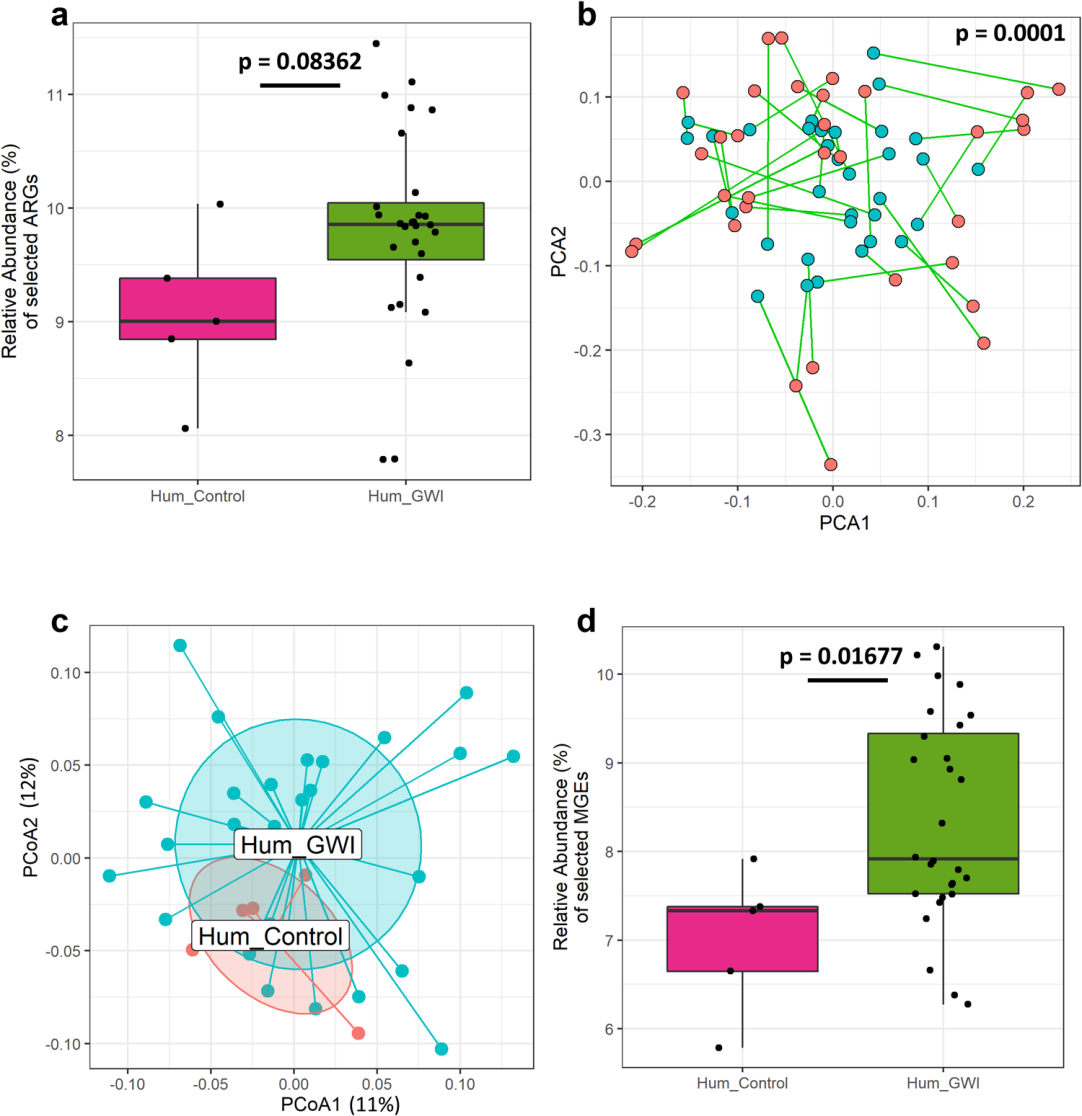
Distribution of ARGs and MGEs in GWI Veteran groups. **a** Box plot showing relative abundance (%) of ARGs in Hum_Control (Veteran group without GWI symptom, *n* = 5 biologically independent samples) and Hum_GWI (Veteran group with GWI symptom, *n* = 28 biologically independent samples). Median values of 9.0% in Hum_Control and 9.86% in Hum_GWI are indicated by solid black lines. **b** Procrustes rotation analysis comparing resistome (blue) and microbiome (red) changes using PCA ordinations, (*X*-axis: PCA1, *Y*-axis: PCA2, PROTEST, *M*^2^ = 0.6665, *p* = 0.0001). **c** PCoA of Bray-Curtis dissimilarity between ARGs and Taxa. Principal coordinate axes PCoA1(12.7%) and PCoA2 (11.5%) account for 24.2% of detected variance. **d** Box plot showing relative abundance (%) of selected MGEs in Hum_Control and Hum_GWI. Boxes in the box plots indicate interquartile range. Error bars in the box plots extend to the most extreme values within 1.5 times the interquartile range. Outlier data points are represented by black dots drawn outside of the box plot. *p*-values were determined using PROTEST or Welch’s *t*-test where *p* < 0.05 was considered statistically significant.

We studied the gut resistome pattern in GW Veterans. We performed whole-genome shotgun sequencing, metagenomic assembly, and functional gene annotation as with the mouse samples. We detected 604 unique ARGs with 224 ± 11 and 238 ± 6 total occurrences in the Hum_Control group and Hum_GWI groups respectively. Comparing the ARG profiles of the two groups, we observed only minor and insignificant deviation in the resistome profiles between the two groups (*p* = 0.2826, PERMANOVA, *R*^2^ = 3.4%). When investigating relative abundance of significantly changed ARGs (DESeq2, negative binomial GLMs, Wald’s test, *p* < 0.05), we observed that the sum relative abundance of selected ARGs increased but not statistically significant in the Hum_GWI group compared to the Hum_Control group (*p* = 0.08362, Fig. 3a). Procrustes analysis showed a significant correlation of bacterial taxa and the ARGs (*p* = 0.0001, PROTEST, *M*^2^ = 0.6665, Fig. 3b). PCoA analysis showed clustering of the Hum_Control and Hum_GWI groups with major overlap on the first two axes accounting for only 24% of the total variance in the dataset (Fig. 3c). Considering the minor divergence indicated by PERMANOVA and the low proportion of explained variance, this result might have stemmed from the increased complexity of the human gut microbiome compared to that of our mouse model.

When studying the transferability, we identified 110 unique MGEs across both groups with 80 ± 5 and 86 ± 2 total MGEs in the Hum_Control group and the Hum_GWI groups respectively. We observed minor variation in the MGE profiles of the two groups which was shown to not be significantly dependent on the sample group variable (*p* = 0.3757, PERMANOVA, *R*^2^ = 3.3%). The relative abundance of selected MGEs were significantly increased in Hum_GWI group compared to the Hum_Control (*p* = 0.01677, Welch two-sample *t*-test, Fig. 3d).

### Distribution of selected ARGs and MGEs in human samples

**Fig. 4:**
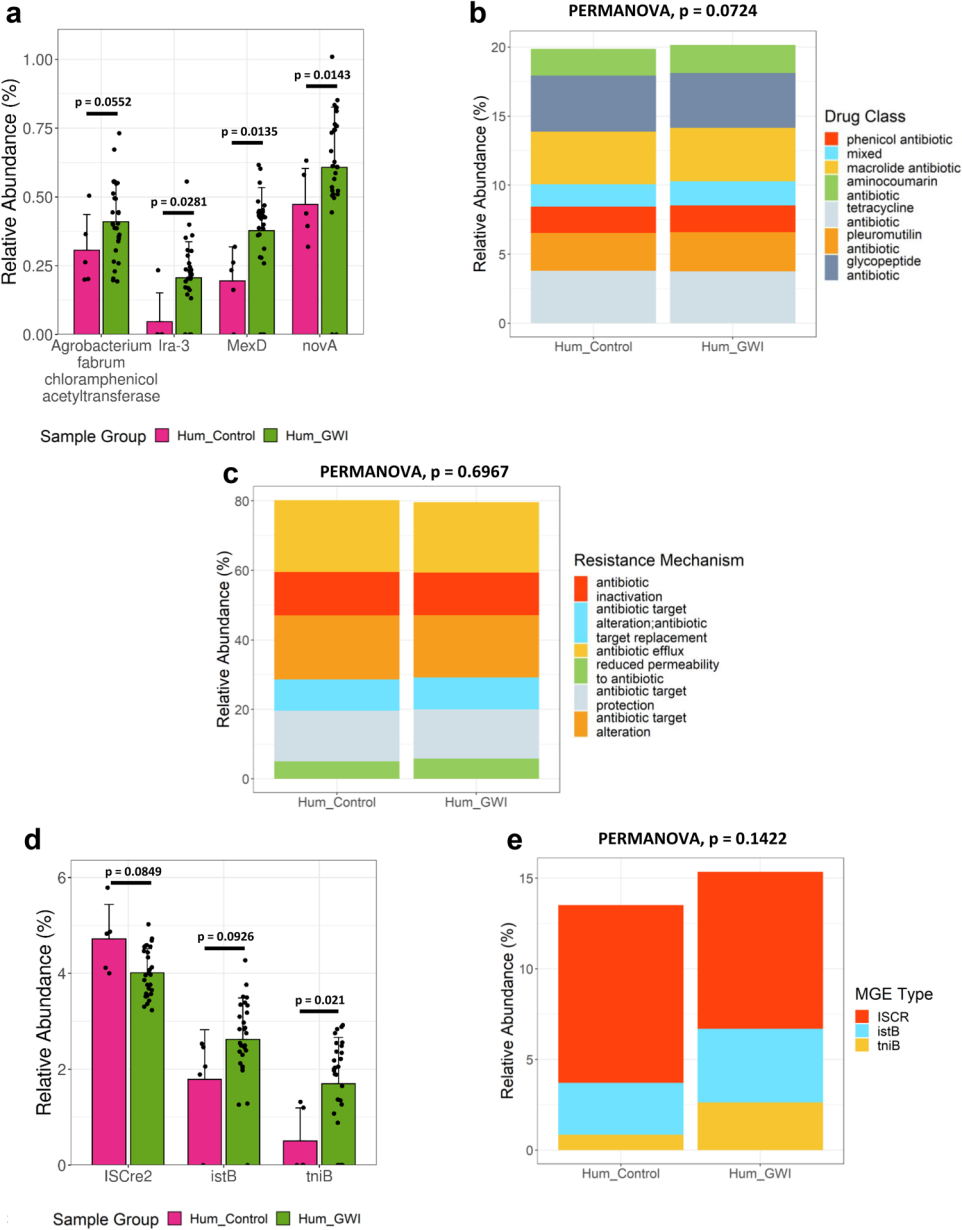
Classification of selected ARGs and MGEs in GWI Veterans groups. **a** Grouped bar graph showing relative abundance (%) of selected ARGs in Hum_Control (Veteran group without GWI symptom, *n* = 5 biologically independent samples) and Hum_GWI (Veteran group with GWI symptom, *n* = 28 biologically independent samples). Stacked bar analysis of relative abundance (%) of **b** drug classes and **c** mechanisms of resistance. **d** Grouped bar graph showing relative abundance (%) of selected MGEs in Hum_Control and Hum_GWI groups. **e** Stacked bar analysis of relative abundance (%) of MGE types. Data represented as Mean±SD (SD: Standard deviation) for the bar graphs. *p*-values were determined using Wald’s test or PERMANOVA where *p* < 0.05 was considered statistically significant.

Our results showed that the relative abundance of individual ARGs was significantly higher in Hum_GWI group compared to Hum_Control group. The glycopeptide resistance gene was found to be the most abundant ARG in both groups of GW Veteran samples (not statistically significant). Based on differential abundance analysis, we found that the abundance of 3 ARGs were significantly increased in the Hum_GWI group compared to the Hum_Control group (DESeq2, negative binomial GLMs, Wald’s test, *p* < 0.05, Fig. 4a).These ARGs were *lra-3* (ARO: 3002510)*, mexD* (ARO: 3000801*)* and *novA* (ARO: 3002522). *Agrobacterium fabrum chloramphenicol acetyltransferase (afca)* (ARO: 3004451) increased in Hum_GWI but it was not statistically significant. These ARGs confer resistance against multiple critically important antibiotics which are used as human medicine (WHO, AGISAR 2018). Furthermore, there were 250 unique ARGs found in Hum_GWI group which were absent in Hum_Control group. Changes in the drug classes and antibiotic resistance mechanism were not statistically significant between the 2 groups (Fig. 4b, c).

The abundance of individual MGEs were also altered in Hum_GWI group compared to Hum_Control group. The MGE *tniB* significantly increased in the Hum_GWI group (*p* = 0.021, DESeq2, negative binomial GLMs, Wald’s test, Fig. 4d). Although a change in the relative abundance of *istB* and *ISCre2* was observed in Hum_GWI group over the Hum_Control group, it was not statistically significant. Among the MGE types, transposons, and most importantly insertional sequence *ISCR* was increased in the Hum_GWI group (not statistically significant, Fig. 4e). *ISCR* has been reported to cause increased mobilization of ARGs among bacteria, presence of this type of mobilome in GW Veterans raises the concern of increased antibiotic resistance^33^. According to our procrustes analysis in GW Veteran samples, changes in the resistome are correlated with the altered gut microbiome, hence increases in MGEs like *ISCR* might increase the chances of ARG mobilization which could be fatal for the GW Veteran health due to increase in resistance to antibiotics. Multivariate analysis of the MGE class profiles revealed minor deviation between sample groups which was not significant (*p* = 0.1422, PERMANOVA, *R*^2^ = 4.5%). Further investigations in GW Veterans are required to prove the resistance against the individual ARGs.

### Expression study of antimicrobial resistance genes in mouse and GW Veteran samples by q-RTPCR analysis

**Fig. 5:**
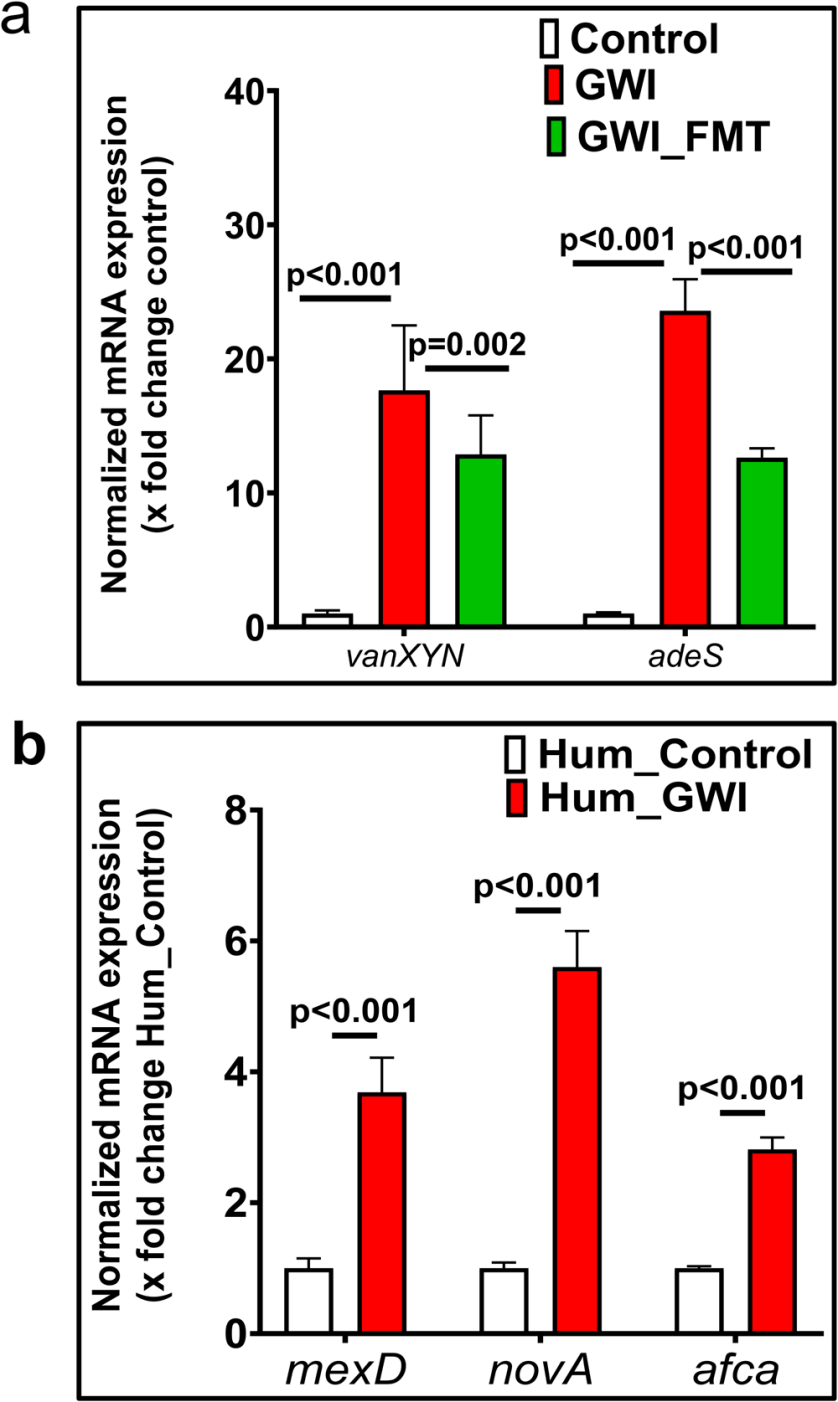
Relative expression levels of antimicrobial resistance genes. Relative mRNA expression of ARGs. **a** Between mouse experimental groups Control (*n* = 11 biologically independent samples), GWI (*n* = 11 biologically independent samples) and GWI_FMT (*n* = 6 biologically independent samples). **b** Between GW Veteran groups Hum_Control (*n* = 5 biologically independent samples) and Hum_GWI (*n* = 28 biologically independent samples). The mRNA expression was calculated as fold change against control (in Fig. 6a) and against Hum_Control (in Fig. 6b). Data are represented as mean ± SEM (SEM:Standard Error Mean). *p*-values were determined using by two-way ANOVA with Bonferroni’s post hoc test for GWI mice groups and GW Veteran groups where *p* < 0.05 was considered statistically significant.

To confirm the expression of the ARGs observed by the gene annotation studies in the previous sections (differential abundance by DESeq2), we performed q-RTPCR analysis of the same ARGs, using DNA extracted from the mouse (Fig. 5a) and GW Veteran fecal samples (Fig. 5b).

In the mouse samples, expression of *vanXYN* was significantly increased by 17.65-fold in the GWI group compared to the Control group (*p* < 0.001, two-way ANOVA with Bonferroni’s post hoc test). Expression of *vanXYN* (12.87 fold compared to the Control group) significantly decreased in the GWI_FMT group compared to the GWI group (*p* = 0.002, two-way ANOVA with Bonferroni’s post hoc test). Similarly, expression of *adeS* was significantly increased by 23.59-fold (*p* < 0.001, two-way ANOVA with Bonferroni’s post hoc test) in the GWI group compared to the Control group. The expression of *adeS* (12.63 fold compared to the Control group) significantly decreased (*p* < 0.001, two-way ANOVA with Bonferroni’s post hoc test) in the GWI_FMT group compared to the GWI group. Although q-RTPCR analysis results showed similar trend like the gene annotation study, i.e., significant increase in expression of the respective ARGs in the mice GWI group and a decrease (statistically significant by q-RTPCR) in GWI_FMT group, further experimental proof would be required to confirm that FMT administration indeed was successful in decreasing the individual ARG expressions.

In the GW Veteran samples, expression of *mexD, novA,* and *afca* was significantly increased by 3.68, 5.59, and 2.81-fold respectively in the Hum_GWI group compared to the Hum_Control group (for *mexD* p, for <0.001 *novA* *p* < 0.001, for *afca* *p* < 0.001 between Hum_Control and Hum_GWI group, two-way ANOVA with Bonferroni’s post hoc test, Fig. 5b).

### Gastrointestinal, systemic, and neuronal inflammation and its association with ARGs and Drug Classes in mouse GWI model samples and GW Veteran samples

**Fig. 6:**
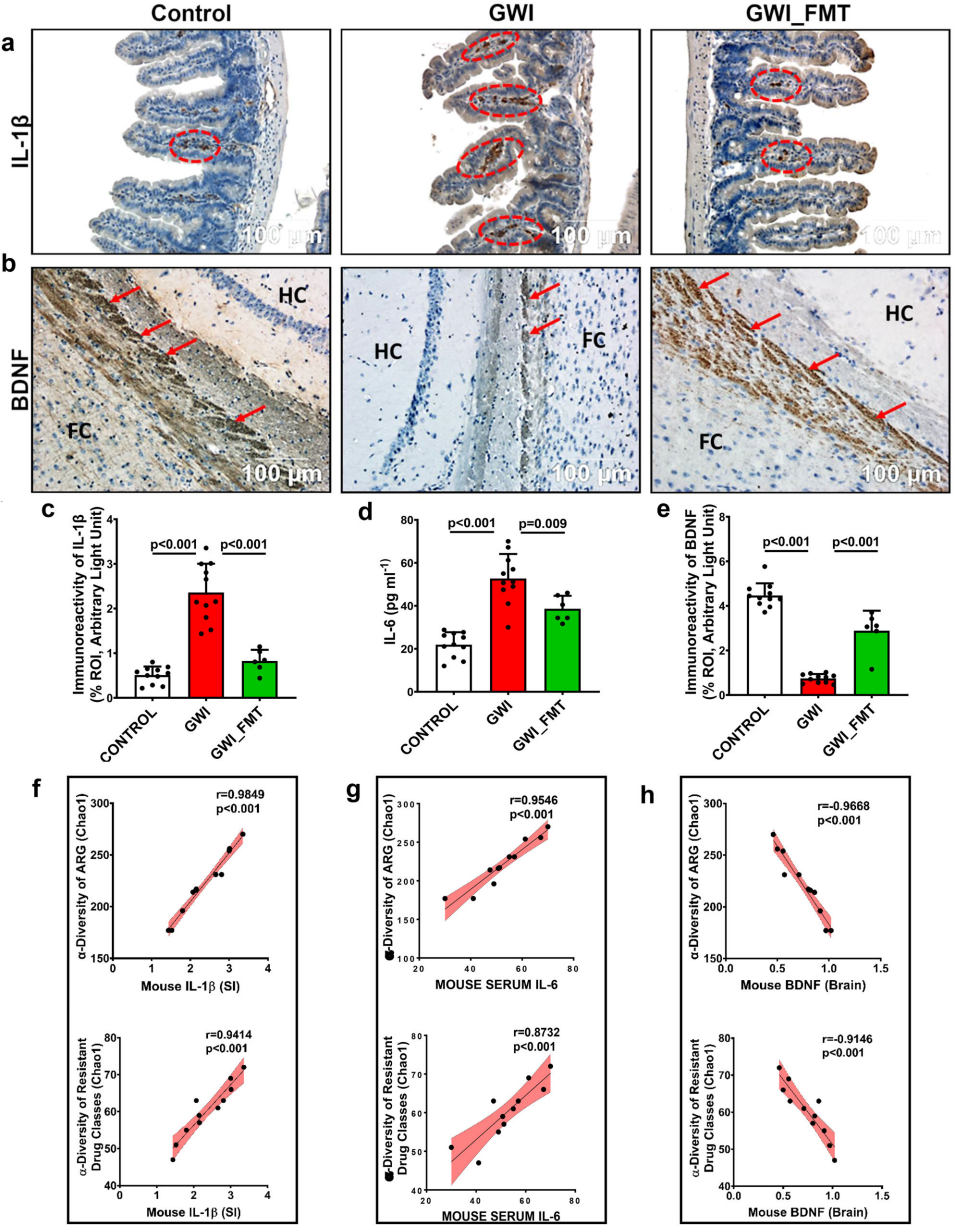
Gastrointestinal, systemic, brain inflammation and its correlation with antibiotic resistance genes and the resistant drug classes in GWI mice model. **a** Representative immunohistochemistry image showing immunoreactivity of proinflammatory cytokine IL-1β (marked by red circle) in mouse experimental groups Control (*n* = 11 biologically independent samples), GWI (*n* = 11 biologically independent samples) and GWI_FMT (*n* = 6 biologically independent samples). Images were taken at 20X magnification. Scale bar = 100 μm **b** Representative immunohistochemistry image showing immunoreactivity of synaptic plasticity marker BDNF (marked by red arrows) in Control (*n* = 11 biologically independent samples), GWI (*n* = 11 biologically independent samples), GWI_FMT (*n* = 6 biologically independent samples) mice groups. Images were taken at 20X magnification. Scale bar = 100 μm. FC Frontal Cortex, HC Hippocampus**. c** Bar graph depicting immunoreactivity of IL-1β. Data are represented as mean ± SD (SD: Standard deviation) of %ROI (mean value calculated from two different fields in each sample). **d** Bar graph depicting the serum IL-6 level at pg/ml in Control, GWI, GWI_FMT mice groups. Data represented as mean ± SD. **e** Bar graph depicting immunoreactivity of BDNF. Results are represented as mean ± SD of %ROI (mean value calculated from 2 different fields in each sample). **f** Correlation plot between α-diversity (Chao1) of resistant ARGs and drug classes and immunoreactivity of IL-1β in mouse GW group in small intestine section. **g** Correlation plot between α-diversity (Chao1) of resistant ARGs and drug classes and serum IL-6 level in mouse GW group. **h** Correlation plot between α-diversity (Chao1) of resistant ARGs and drug classes and immunoreactivity of BDNF in mouse GW group in brain section. *r*-value was determined by Pearson’s Regression analysis. Pearson’s linear regression is shown in red with 95% confidence bands. Statistical significance was analyzed by one-way ANOVA (Tukey’s post hoc test) where *p* < 0.05 was considered statistically significant.

Gastrointestinal and neuronal inflammation is reported in GW Veterans and in preclinical GWI mice models due to the influence of GW chemicals^31,34–38^. Increases in systemic inflammatory markers were also reported in GW Veterans and mouse models^31,38–40^. Following our study of the abundance and expression of ARGs and MGEs, we sought to identify a link between ARGs and biomarkers of GWI pathology which would aid in establishing a role of gut resistome in influencing host health. The purpose of this study was also to show whether a predictive insight can be made regarding future susceptibility to infectious diseases related to hospital acquired infections in GW Veterans, elderly, and immunocompromised individuals. Results in mice showed that the expression of IL-1β in the small intestine significantly increased in GWI group (*p* < 0.001, one-way ANOVA with Tukey’s post hoc test) compared to the Control group, as shown through immunoreactivity of the cytokine in the villi (Fig. 6a, c). Treatment with FMT significantly decreased the expression of IL-1β in the GWI_FMT mice group compared to the GWI group (*p* < 0.001, one-way ANOVA with Tukey’s post hoc test, Fig. 6a, c). To study the association between IL-1β expression and ARG diversity, we performed a correlation analysis. Results showed a positive correlation (*r* = 0.9849, *p* < 0.001 and *r* = 0.9414, *p* < 0.001 respectively, Pearson regression analysis) between α-diversity of ARGs, resistant drug classes and increased IL-1β in GWI mice group, suggesting that alteration of the gut resistome profile had a significant association with gastrointestinal inflammation (Fig. 6f).

We observed a significant increase in serum IL-6 level in the GWI mice group when compared to the Control. Further, there was a significant decrease in serum IL-6 levels in GWI_FMT groups when compared to GWI group (*p* < 0.001 between GWI and Control, *p* = 0.009 between GWI and GWI_FMT, one-way ANOVA with Tukey’s post hoc test, Fig. 6d). We also observed that α-diversity of ARGs and resistant drug classes were positively correlated (*r* = 0.9546, *p* < 0.001 and *r* = 0.8732, *p* < 0.001, Pearson regression analysis) with increased systemic IL-6 level in the GWI mice group (Fig. 6g).

Our previous studies have shown that a decrease in synaptic plasticity marker brain derived neurotrophic factor (BDNF) played a key role in brain pathology in GW chemical exposed mice^41^. Results showed that the expression of BDNF significantly decreased in GWI group when compared to Control. Further, the levels of BDNF as determined by morphometry from immunohistochemical analysis showed that there was a significant increase in the BDNF levels in the GWI_FMT mice groups when compared to GWI group (*p* < 0.001 between Control vs GWI; *p* < 0.001 between GWI vs GWI_FMT respectively, one-way ANOVA with Tukey’s post hoc test, Fig. 6b, e). Interestingly, a negative correlation was observed between BDNF and ARGs and resistant drug classes (*r* = −0.9668, *p* < 0.001 and *r* = −0.9146, *p* < 0.001 respectively, Pearson regression analysis) suggesting that increased ARG-α-diversity may have a strong influence on observed neuroinflammation in the GWI mice group (Fig. 6h).

**Fig. 7:**
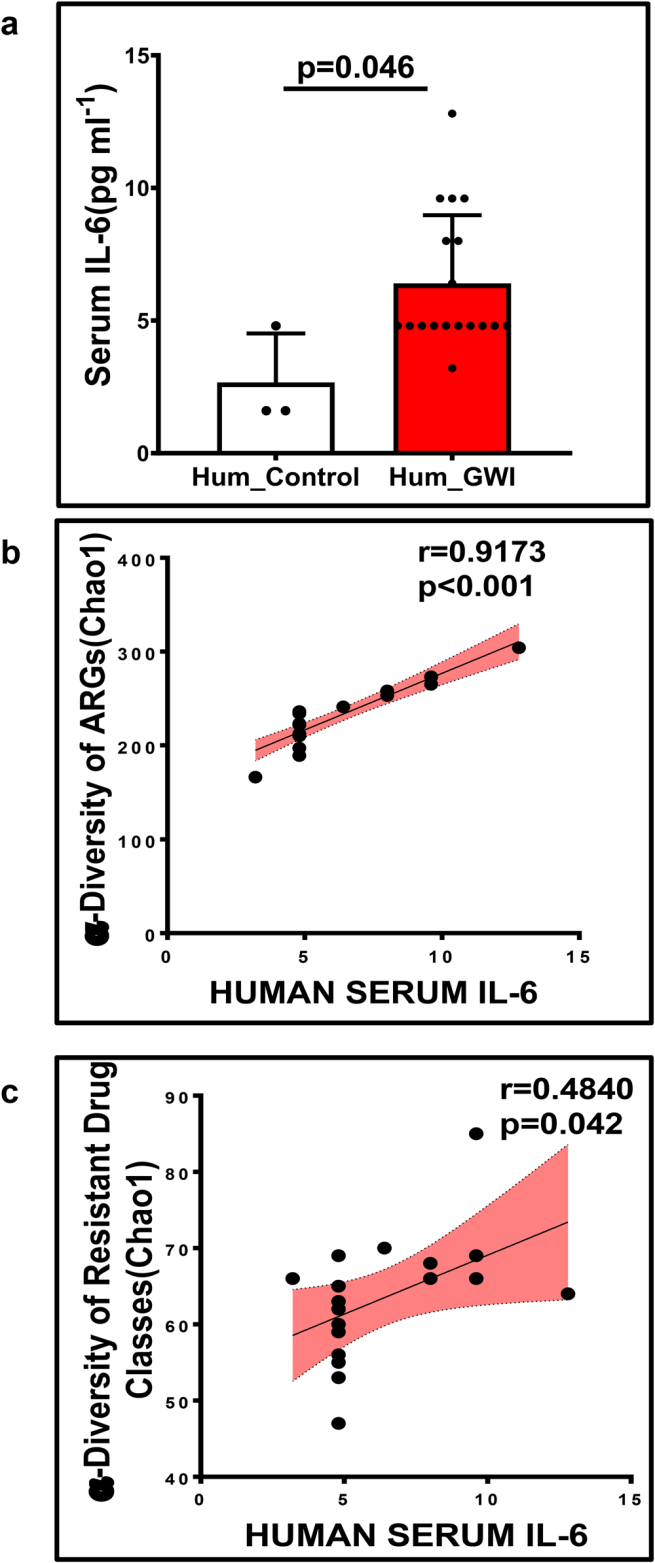
Systemic IL-6 level and its correlation with antibiotic resistance genes and the resistant drug classes in GW Veteran samples. **a** Bar graph depicting the serum IL-6 level at pg/ml in Veteran control group Hum_Control (*n* = 3 biologically independent samples) and Hum_GWI (*n* = 18 biologically independent samples) groups. Correlation plot between α-diversity (Chao1) of **b** resistant ARGs and **c** resistant drug classes and serum IL-6 in Hum_GW group. Data are represented as mean ± SD (SD standard deviation). *r*-value was determined by Pearson’s regression analysis. Pearson’s linear regression is shown in red with 95% confidence bands. Statistical significance was analyzed by Welch’s two-tailed *t*-test where *p* < 0.05 was considered statistically significant.

Furthermore, we studied the level of systemic IL-6 in the serum collected from the respective GW Veterans in this study. Results showed a significantly increased level of serum IL-6 in the Hum_GWI group compared to the Hum_Control group (*p* = 0.046, Welch’s *t*-test; Fig. 7a). Correlation analysis between serum IL-6 level and the α-diversity of ARGs and resistant drug classes (*r* = 0.9173, *p* < 0.001 and *r* = 0.4840, *p* = 0.042 respectively, Pearson regression analysis) in GW Veterans showed a positive correlation (Fig. 7b, c). This results may be suggestive of a link between altered gut resistome and increased systemic IL-6 in the human samples. However, further experimentation is required to confirm this hypothesis.

## Discussion

In the present study, we report an alteration of host gut resistome in chronic multisymptom illness as observed in a subsection of Gulf War Veterans. We also report the association of systemic inflammation with an altered gut resistome that in turn is further correlated with increased circulating proinflammatory cytokine IL-6, intestinal pathology and neurotrophic factor BDNF in preclinical GWI mice model. Most importantly, we have shown that increased systemic IL-6 had strong association with altered human gut resistome in GW Veterans. IL-6 has been shown to be a pleiotropic cytokine and is key to gastrointestinal disturbances, and cognitive deficits^42,43^. Recent advances in understanding the host resistome improved our knowledge about evolution, origins and emergence of antibiotic resistance though the field is continuously evolving^44^. Previously, our knowledge of resistome included proto-resistance and silent-resistance genes^44^. Silent-resistance genes do not cause phenotypic resistance until they are transferred via MGEs, or a mutation occurs in the associated regulatory elements. Gut microbiome is usually associated with a silent resistome, as they have the potential to contribute in clinical resistance through mobilization^44^. Our study supports the existing evidence that the resistome is highly dependent on the gut microbiome and indicates to a possible association between changes in the bacteriome and the gut resistome, particularly in GWI groups, both in the case of mouse models and Veterans with GWI as studied by PERMANOVA and procrustes analysis (Figs. 1b and 3b).

Exposure to environmental chemicals like pesticides, biocides, aerosols have been reported to the increase in antimicrobial resistance via HGT which have long-lasting changes and increase in these resistant bacterial population in human due to selection pressure leads to treatment failure with available antibiotics^12,16,17,19,45–48^. This led us to our hypothesis that exposure to a mixture of environmental hazards (war theater) in GW Veterans as well as representative GW chemical exposure in GWI mice model may affect the gut resistome. Our results suggested that representative GW chemicals PB and permethrin (Per) may change the ARGs significantly, but the changes observed in individual MGE profiles in GWI group of the mouse model when compared to the control group were not significant. One of the limitations in this study includes our inability to experimentally confirm that the GW chemical-treated mice were indeed resistant to the classes of antibiotics or the individual ARGs that has been found in this study. However, we would also like to state that to the best of our knowledge no GWI mice model could exactly mimic the health condition of the GW Veterans. Different combinations of GW chemicals along with different routes of administration needs to be included during the various GWI mice model studies to further elucidate the mechanisms proposed. Future studies need to overcome these limitations for in-depth mechanistic links to gut resistome in GWI mice models.

Previous reports stated that elderly individuals and Veterans had increased resistance to sulfonamide, macrolide, β-lactam antibiotics followed by tetracycline as well as fluoroquinolones especially found in *Acinetobacter baumannii* isolates^49,50^. Reports also stated that fluoroquinolones and cephalosporin usage should be prescribed in a limited manner among the elderly and Veterans as they have higher susceptibility in developing resistances upon treatment with such high generation antibiotics^50^. However, there is very little evidence about the ARGs in Veterans, especially GW Veterans suffering from CMI or collectively referred as GWI who were deployed 30 years back and are presently in the age range of 50–60 years. The present study attempted to report the alteration of ARG and MGE signature in GW Veterans belonging to the aforementioned age group. Another limitation of the present study includes the inability to confirm whether the gut resistome alteration is a result of GW chemical exposures alone. GW Veterans may have been exposed to multiple courses of antibiotics at different points in their lifetime, along with this factors like diet, metabolic conditions like obesity that might prompt an increase in the altered gut resistome. A further limitation in our analysis of gut resistome in GW Veterans may be whether the GW Veterans recruited in our study were actually resistant to the ARGs and antibiotics reported. The limitation is also confounded by the small sample size of Veterans that may have caused low significance of our ARG and MGE results. To overcome these limitations, future studies with a large cohorts with sufficient power would help to obtain more in-depth knowledge about the antibiotic resistance in GWI Veterans. We identified that glycopeptide resistance was high in GWI compared to other mouse groups as well as in GW Veterans (not statistically significant). Glycopeptide group of antibiotics especially Vancomycin is used to treat bacterial infections like methicillin-resistant *Staphylococcus aureus* (MRSA)*, Clostridium difficle,* and *Enterococcus spp*.^51^. Recently it has been reported that administration of Vancomycin in patients at Veterans Affair Hospitals have been ineffective against these clinically important bacteria and causing nephrotoxicity^52–55^. Increased glycopeptide resistance as detected in our GW Veteran samples might be suggestive of the reason behind the ineffectiveness of Vancomycin antibiotic used to treat a section of Veterans. A further study of the resistant drug classes as well as mechanisms of resistance could be helpful to confirm this result.

FMT has been reported to restore the beneficial microbiome in pathological conditions like inflammatory bowel disease (IBD), type 2 diabetes, nonalcoholic steatohepatitis and neurological disorders where gut microbial dysbiosis plays a direct role in the disease progression^56–62^. Studies have also reported that FMT administration have decreased the ARGs in patients with cirrhosis and *Clostridium difficile* infection^62^. In the present study, we aimed to study if FMT could be a therapeutic by decreasing antimicrobial resistance in GWI condition in preclinical GWI mice model. We observed a decrease in relative individual ARGs and MGEs in GWI_FMT group compared to GWI group in the GWI mice model (Fig. 2a, d). FMT treatment was able to significantly ameliorate GW chemical induced gastrointestinal inflammation and systemic inflammation and significantly increase synaptic plasticity marker expression. Moreover, future dosing considerations are required in FMT treatment since prolonged FMT treatment may cause stress alteration of gut microbiome and resistome. This also emphasizes the need for screening the fecal samples of healthy donors for FMT which has been a limitation^63^. Hence, these criteria should be considered in using FMT as a therapeutic measure to ameliorate gut resistome alteration and systemic inflammation in GW Veterans through carefully designed clinical trials.

Interestingly, we observed the accumulation of ARGs that are highly mobile and transferable. The mice in the preclinical GWI model were never exposed to any classes of antibiotics during the entire course of the study, but they showed a spontaneous acquisition of unique ARGs in GW chemical exposed group but not in Control and GWI_FMT groups. This transfer may occur due to intrinsic or extrinsic transfer by HGT method based on MGE patterns. This study also suggested that GW chemical exposure may be responsible for the alteration and appearance of ARGs and MGEs in mice since they have been maintained in a controlled environment with very limited risk of exposure to a multifaceted environment, though the presence of these ARGs needs to be further elucidated.

We were able to identify diverse types of MGEs in which transposons were majorly observed in both mouse and GW Veteran samples. A study by Parnanen et al. stated that transposase constitutes the most abundant MGE class^20^. This study completely is consistent with our observations about MGEs. Higher abundance of MGEs belonging to transposon group along with integrons, insertional sequence might indicate a probability for inter-bacterial ARG transfer which might increase the adverse consequences due to increase in antimicrobial resistance in GWI pathology^9,64,65^. To track the ARG transfer through MGE, further detailed investigation is needed to identify the ARGs associated with the MGEs.

Studies have also reported that low dose antibiotics have increased the abundance of a single pathogenic bacteria, however, difference in ARGs were not significant compared to control groups^66^. This study also showed change in immunological markers but association between ARGs and immunological markers have not been established^66^. Also, IBD phenotype is known to be associated with microbiome dysbiosis which led to upregulation of antibiotic resistance^67^, a condition also observed in GW Veterans^68^. We have shown an association between gastrointestinal, systemic, and neuronal inflammation observed in GWI pathology and diversity of ARGs and resistant drug classes in a GWI mice model. Our results also indicated an association between systemic inflammation reflected by increased serum IL-6 and ARG diversity in GW Veterans. Interestingly, association between diversity of ARGs and systemic proinflammatory marker IL-6 will be an important benchmark in future studies of chronic multisymptom illness and other related pathology. Future mechanistic studies are needed to establish the exact role of ARGs in GWI pathophysiology by using germ free and gnotobiotic models combined with the use of short chain fatty acid treatment regimens such as butyrate therapy.

### Limitations of the study

Modeling the chronic multisyptomatic condition of GWI in rodent models to mimic the health condition of the present-day GW Veterans has been challenging. Due to the complex environmental chemical exposures during the Persian Gulf War, it is imperative that incorporation of other chemicals like organophosphates, N,N-diethyl-meta-toluamide (DEET), depleted uranium exposure, vaccines, oil smoke along with Per and PB would strengthen the translatability of the results in GWI. The research in GWI should consider the differences in the routes of exposure of these environmental mixtures such as the dermal route for administration of Per, a widely used insecticide. Small sample size of the GWI persistence mouse model as well as GWI Veteran cohorts may have affected the resistome analysis, hence an incorporation of a larger sample size along with studies with significant statistical power in future mechanistic analyses of the gut resistome will help us in better understanding the changes ARGs and MGEs. This is especially important in detecting the minute changes related to the resistant drug classes, antibiotic resistance mechanisms and MGE types. In the GWI mice model, inclusion of an FMT-only control would help us to clearly distinguish the effects of the above treatment regimen on gut resistome. A modification of the treatment plan of FMT in the murine model that takes into account the additional stress-induced changes in the gut resistome that might have occurred, would be pertinent to the present line of investigation. Future studies would also need to screen the fecal samples from the healthy mice-Control groups to minimize the transfer of ARGs to the recipient group. The present study should not be conceived as an attempt to integrate the mouse data with that of the data from the human cohorts as there are remarkable differences in the exposure patterns as underlined in our previous sections. The human cohort was included to investigate if resistome alteration occurred in GWI conditions as an effect of microbiome dysbiosis, as reported in earlier studies. The GW Veterans have been exposed to multiple environmental chemical exposures singly or in combination. In addition, diet, sedentary lifestyle after deployment, metabolic conditions like obesity and exposure to multiple courses of antibiotics and treatment regimens may have contributed to higher resistome diversity. Another significant limitation might be the inability to confirm experimentally if the GWI Veterans or the mice exposed to representative GWI chemicals were actually resistant to the antibiotics observed from the gene annotation studies. These limitations could have been overcome by exposing GWI mice to representative antibiotics following a bacterial infection or an acute challenge with bacteria that cause pathogenesis. Inclusion of these set of experiments might have supported our hypothesis that a resistome signature can predict phenotypic disease susceptibility later in life. Also, inclusion of the previously stated factors like diet and obesity in the future GWI mouse model study along with use of humanized and germ-free mice might improve the translatability of the results obtained from the animal studies. We were also limited in linking the MGEs with the ARGs as well as identifying the mechanism that connected the GWI pathology with altered gut resistome which we plan to study in the immediate future. The present study is a preliminary report. The observations can help build a stronger hypothesis that points to the role of the host gut resistome in GWI disease pathology. Further studies with larger sample sizes may establish causality and help in determining risk of GWI Veterans to antibiotics and other co-morbidities.

In conclusion, to the best of our knowledge the present study is the first to investigate gut resistome alterations in GWI. Further, a preliminary association was established between an altered resistome and systemic IL-6 levels in a translatable mouse model that has broad implications in the general population suffering from similar ailments though the actual causality is yet to be established. It is expected that 78 million of the US population is expected to be in the elderly category by 2030. Most of them have a history of prolonged antibiotic use, a case similar with our aging Veterans. The elderly population belonging to the age group of 50–60 years have increased risk of acquiring antibiotic resistance due to several factors like impaired immune functioning, immunosenescence, and exposure to muti-drug resistant bacteria due to multiple visits to clinics and hospitals^69–71^. In the present study, the GW Veterans were of the same age group (53–56 years) and studies have reported that the present day GW Veterans have a maladaptive immune system primarily due to GW chemical exposure and aging^72,73^. The scenario is also significant owing to old age associated hospitalizations and increased chances of hospital acquired infections. Based on the above facts and our study results, we can predict that GW Veterans may have a very high chance of acquiring antimicrobial resistance. In addition, FMT can be used as a therapeutic strategy against the increased antibiotic resistance in Veterans and elderly to attenuate a possible altered resistome. Knowledge gained from the microbiome and resistome profiles from GW Veterans can be very helpful for treating a variety of bacterial diseases or hospital acquired infections (following surgery) that may require antibiotic treatment in the Veterans and can be a useful tool for a personalized medicine approach.

## Methods

Per and PB were purchased from Sigma-Aldrich. Primary antibodies anti-interleukin-1β (IL-1β), anti-brain derived neurotrophic factor (BDNF) were purchased from Santacruz Biotechnology (Dallas, TX, USA). Species specific biotinylated secondary antibodies and streptavidin-HRP (Vectastain ABC Kit) were purchased from Vector laboratories (Burlingame, CA, USA). All other chemicals used in the present study were purchased from Sigma unless specified. Animal tissues were sent for paraffin embedding and sectioning to AML Laboratories (St. Augustine, FL, USA). Fecal samples from experimental mice groups and GW Veterans were sent to COSMOSID (Germantown, MD, USA) for whole-genome sequencing.

### Animals

C57BL/6 J wild type, male mice of 10 weeks age were purchased from Jackson Laboratories (Bar Harbor, ME, USA). The mice were maintained in accordance with local IACUC standards and National Institute of Health guidelines for human care and use of laboratory animals. All animal experimental procedures were approved by University of South Carolina at Columbia, SC (Animal protocol number 2419-101345-072318, approved on 7/23/2020). All the mice had ad libitum access to food and water and were housed at 22–24 °C with 12 h light/12 h dark cycles. The mice were sacrificed after the animal experiments. Organs including frontal cortex and distal part of small intestine were collected after dissecting the mice and fixed in Bouin’s solution and 10% neutral buffered formaldehyde respectively. Serum was collected from fresh blood of mice by performing cardiac puncture after anesthesia. The fecal pellets were collected from colon, and it was stored at −80 °C for whole-genome sequencing (WGS).

### Mouse model of Gulf War illness

**Fig. 8:**
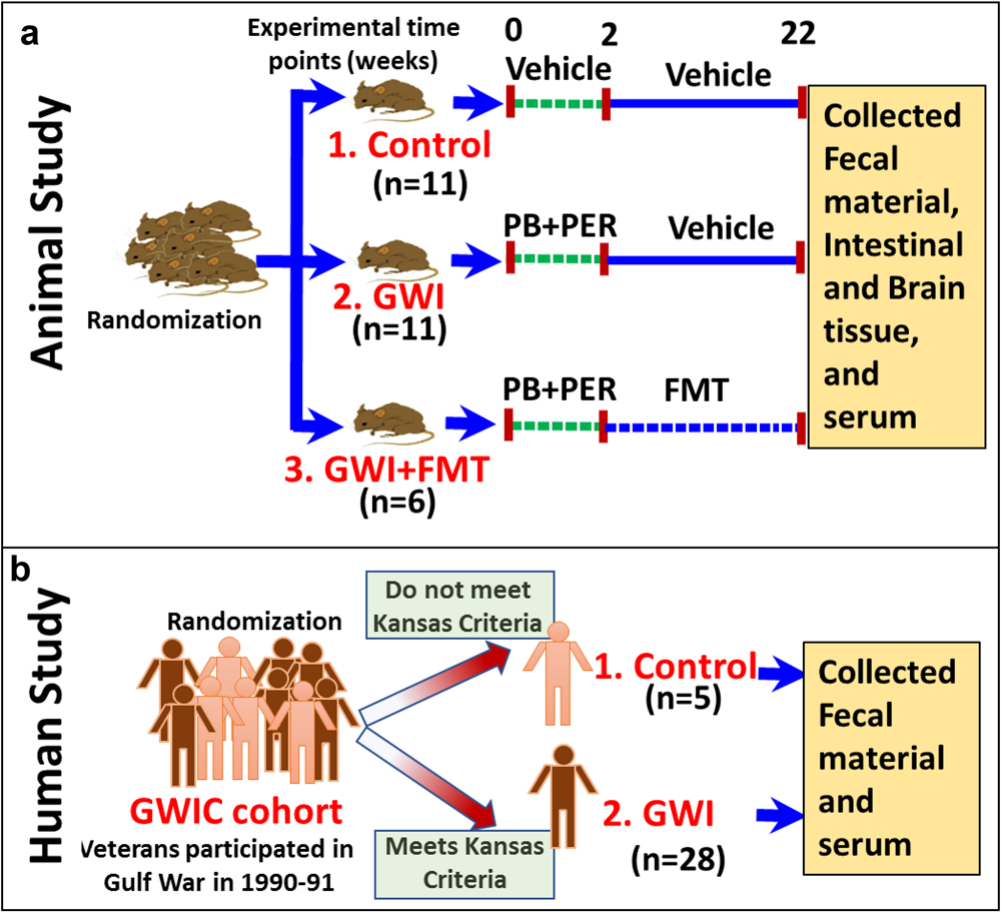
A schematic representation of the experimental cohorts. **a** Experimental mouse model of Gulf War Illness (GWI). **b** Study design of Veteran participants in Boston Gulf War Illness Consortium (GWIC) based on Kansas GWI criteria.

After 1 week of acclimatization, the mice were randomly distributed into three groups. The first group received vehicle (0.6% dimethyl sulfoxide) for 2 weeks and were denoted Control (*n* = 11). The second and third mice groups denoted GWI (*n* = 11) and GWI_FMT (*n* = 6) were treated with Per [200 mg/kg body dissolved in DMSO and phosphate buffer saline(PBS)] and PB (2 mg/kg dissolved in PBS) by oral gavage tri-weekly for 15 days. After the 2 weeks of GW chemical exposure, GWI group mice were allowed to persist for 20 weeks. Fecal microbiota transplant was administered in GWI_FMT after GW chemical exposure. 100 mg of fecal pellets were collected from healthy C57BL/6 J mice of same age group as the GWI_FMT mice. The pellets were homogenized in 1 ml of PBS and centrifuged at 3000 × *g* for 5 min. 100 μl of supernatant was dosed in each mouse on alternate days of a week for 20 weeks^74–76^ (Fig. 8a).

### Human subjects: GW Veterans with GWI and controls

The Boston Gulf War Illness Consortium (GWIC) performs preclinical and clinical studies to understand the pathophysiology behind the complex symptoms in GW Veterans to aid in designing of possible therapeutic strategies^77^. Veterans were included as participants in GWIC studies based on requirement that they had to be deployed in the GW i.e., from August 1990 to July 1991. The GWIC used the Kansas GWI criteria as the case definition which requires the Veterans to have symptoms in 3 out of 6 broadly defined group of symptoms (neurological, pain, gastrointestinal, skin, respiratory, fatigue) to meet criteria of CMI in GW Veterans known as Gulf War Illness (GWI)^31,78^. GW Veterans who do not meet Kansas criteria are deemed the control group.

The Veterans who participated in the GWIC, underwent multiple tests including neuropsychological assessments, health surveys, biological specimen collection, and brain imaging^27,77^. The present study was conducted as a GWIC call-back study in which we aimed to reassess 150 of the GWIC participants. For this study, data from the first 33 recruited subjects from the microbiome call-back study has been analyzed. The recruitment of participants was via telephone on completion of GWIC study protocol. After filling out a brief questionnaire regarding screening, the participants were sent a stool collection kit which was then shipped back to the study investigators (Fig. 8b).

### Collection of demographical, deployment exposure, and health symptom information from GWIC participants

The full GWIC protocol has been previously published in Steele et al., 2021^77^. Briefly, the GWIC participants had to answer to surveys regarding demographics and health condition which included Multi-dimensional Fatigue Inventory (MFI-20), Pittsburg Sleep Quality Index and McGill Pain Inventory^79–81^. The Structured Neurotoxicant Assessment Checklist (SNAC) and Kansas Gulf War and health Questionnaire and Kansas Gulf War Experiences surveys were given to obtain details about self-reported exposures. The survey regarding health condition provided the details if the participants had an ascertained diagnosis of the medical conditions reported by them^31,78,82^. As part of the call-back study, the Veterans also filled out questionnaires about their current and recent gut health and use of antibiotics or probiotics.

### Collection of stool samples from participants

The GWIC participants of the microbiome study were mailed a Second Genome stool collection kit (Second Genome, San Francisco, CA, USA). The kit was a self-collecting kit which contained a bar-coded vial with stabilizing solution for long term preservation of nucleic acids in stool during transportation and storage. Once received from the subjects, the stool samples were stored at −20 °C and upon collection of significant sample numbers, they were sent for WGS by COSMOSID. The protocol was approved by Institutional Review Board at Boston University School of Public Health (proposal no. GW170068) on 4/15/2021. Sample collection from GWIC participants were done following all ethical guidelines and informed consent was also obtained.

### DNA extraction and whole-genome shotgun sequencing

Briefly, the total DNA from mouse and human samples were isolated and purified using ZymoBIOMICS Miniprep kit. DNA was quantified using Qubit dsDNA HS assay (Thermofisher, Waltham, MA, USA). Illumina Nextera XT library preparation kit was used with modifications for preparing DNA libraries. Illumina HiSeq 4000 and Illumina NextSeq 550 platform was used to perform WGS for mice and human samples respectively, following protocol optimized by vendor. 2x150bp of read length and an average insert size of 1400 bp were used for sequencing. DNA libraries were prepared using the Nextera XT DNA Library Preparation Kit (Illumina) with Nextera Index Kit (Illumina) for mice and IDT Unique Dual Indexes for human samples, with total DNA input of 1 ng. Genomic DNA was fragmented using a proportional amount of Illumina Nextera XT fragmentation enzyme. Combinatory dual indexes were added to each sample followed by 12 cycles of PCR to construct libraries. DNA libraries were purified using AMpure magnetic Beads (Beckman Coulter) and eluted in QIAGEN EB buffer. DNA libraries were quantified using Qubit 4 fluorometer and Qubit™ dsDNA HS Assay Kit. Upon data arrival, raw data were backed up to Amazon AWS and run through fastqc and a multiqc report was generated^83^ The multiqc report was checked to ensure read depth thresholds were met, and that there were no abnormalities with read quality, duplication rates, or adapter content. Taxonomic results were checked on the COSMOSID-Hub Microbiome platform to ensure there were contamination or barcoding issues.

We obtained a total of 536.92 million (M) sequencing reads with per sample averages of 9.33 M reads in the Control samples, 8.49 M reads in the GWI samples, 10.12 M reads in the GWI_FMT samples, 11.32 M reads in the Hum_Control samples, and 11.17 M reads in the Hum_GWI samples.

### Metagenomic analysis and assembly

MetaPhlAn v3.0.7^84^ was used to profile the taxonomic composition of each sample with default parameters. The resulting relative abundance tables were then merged with the provided python tool, “merge_metaphlan_tables.py”. A custom python script was used to filter the data to contain only species level identifications and prepare the operational taxonomic unit (OTU) table for statistical analysis. The metaWRAP v1.3.2^85^ pipeline was used to process and assemble raw sequencing reads from each sample. First, the “read_qc” module was used with default parameters to trim sequencing adapters and bases with low PHRED scores. To decontaminate the data, reads mapping to the human reference genome GRCh38.p12 (RefSeq Acc: GCF_000001405.38) and the mouse reference genome GRCm38.p6 (RefSeq Acc: GCF_000001635.26) were removed by the metaWRAP “read_qc” module. After decontamination and quality control, we recovered a total of 532.43 million (M) sequencing reads with per sample averages of 9.22 M reads in the Control samples, 8.24 M reads in the GWI samples, 9.96 M reads in the GWI_FMT samples, 11.28 M reads in the Hum_Control samples, and 11.13 M reads in the Hum_GWI samples. Decontaminated reads were used for de novo assembly using metaSPAdes^86^ as contained in the metaWRAP “assembly” module with default parameters. Resulting contigs were binned by the “binning” module which uses three binning methods, metaBAT2 v2.12.1^87^, MaxBin2 v2.2.6^88^, and CONCOCT v1.0.0^89^ to produce three sets of bins. The “bin_refinement” module was used to refine these three bin sets to produce a single set of best bins. Finally, the single bin set was used by the “bin_reassembly” module which extracts the reads mapping to each bin and uses them for a second round of de novo assembly to improve the completion and reduce the contamination of the bins.

### Antimicrobial resistance gene family and MGE identification

Contigs produced through metaWRAP were used for open reading frame (ORF) finding using MetaProdigal v2.6.3^90^ with parameters “-c -p meta”. ORFs were then clustered with CD-HIT v4.8.1^91,92^ with parameters “-c 0.95 -s 0.90” corresponding to 95% sequence identity threshold over 90% of the shorter ORF length. Next, ORFs were mapped to the Comprehensive Antibiotic Resistance Database (CARD) v3.1.0^93^ and a recently published custom Mobile Genetic Element (MGE) database composed of 278 distinct genes and over 2000 unique gene sequences^20^ using the tool “nhmmer” from the HMMER v3.3.1^94^ software with parameters “-E 0.001 -incE 0.001” corresponding to an *e*-value threshold of 0.001 for matches. A custom python script was used to filter multiple hits to select the single best hit for each ORF. Finally, Microsoft Excel was used to generate count data for ARGs and MGEs.

### Laboratory methods

#### Immunohistochemistry

The fixed mouse small intestine and frontal cortex tissues were paraffin embedded and 5 µm thick sections were done for immunohistochemistry. Deparaffinization were performed following previous protocol^41^. Antigen retrieval was performed using epitope retrieval solution and steamer (IHC world, Woodstock, MD, USA). Three percent hydrogen peroxide was used for blocking endogenous peroxidase activity for 20 mins. Serum blocking was performed using 10% goat serum for 1 h. Tissue sections were incubated with primary antibodies for IL-1β and BDNF at 1:200 dilution for overnight in humified chamber at 4 °C. After incubation, the tissue sections were washed three times with PBS containing 0.05% Tween 20 solution. Tissues were probed with species specific biotinylated antibodies (1:200 dilution) followed by incubation with horse radish conjugated streptavidin (1:500 dilution). 3,3'-diaminobenzidine was used as chromogenic substrate solution and counterstaining was performed using Mayer’s hematoxylin. The stained tissues sections were mounted using Aqua Mount (Lerner Laboratories, Kalamazoo, MI, USA). The images were acquired using Olympus BX63 microscope (Olympus, Center Valley, PA, USA). Morphometry was performed using Cellsens Software from Olympus America (Center Valley, PA, USA).

#### Quantitative RT-PCR

Quantitative RT-PCR (qRT-PCR) was performed to measure ARG expression in DNA extracted from mouse and human stool samples. Gene specific primers were designed using Primer3 (version 0.4.0) and IDT, purchased from Sigma (St. Louis, MO, USA) (Supplementary Table 1). SYBR Green Supermix (BioRad, Hercules, CA, USA) was used in CFX96 thermal cycler (BioRad, Hercules, CA, USA). The samples (both mouse and human) were run in triplicates for each gene. Ct or threshold cycle values of all ARG genes were normalized with 16 S as internal control. 2^−ΔΔct^ method was used to calculate the relative fold change of the ARGs.

#### ELISA

Serum IL-6 level was estimated using serum collected from the mice groups using commercially available kit from Proteintech (Rosemont, IL, USA). Serum IL-6 of Veteran participants in this study was measured using commercially available ELISA kit from Abcam (Boston, MA, USA). The ELISA was performed according to the manufacturer’s protocol.

#### Statistical analysis and reproducibility

Analyses were performed using R v3.6.3 (1). ARG, MGE, and OTU count data were normalized based on library size using the “estimateSizeFactors” function from the “DESeq2” package^95^ with the parameters “type = poscounts”. Normalized count data were then log transformed with the base R function “log2”. Permutational analysis of variance (PERMANOVA) was calculated using the “adonis” function from the package “vegan” (2) with Bray-Curtis dissimilarity and 9999 permutations. Welch two-sample *t*-test was implemented using the base R function “t.test” with the parameters “conf.level = 0.95, alternative = two.sided” indicating 95% confidence level and two-tailed testing. PCA ordinations of ARG and Taxonomy abundance data were performed using the “rda” function from “vegan”. Procrustes analysis was performed using PCA ordinations with the function “protest” from “vegan” with 9999 permutations. Principle coordinate analysis (PCoA) was performed with the “pcoa” function from the “ape” package with Bray-Curtis dissimilarity. Statistical analysis for q-RTPCR, immunohistochemistry and ELISA was done using Prism (Graphpad, San Diego, CA). One-way ANOVA was used for comparing between the three mice groups and Welch’s *t*-test was used for comparison between the Veteran groups for statistical analysis of immunohistochemistry and ELISA. Chao1 α-diversity was calculated using the “chao1” function from the “fossil” package. Correlation analyses between α-diversity and selected biomarkers were performed using Pearson’s correlation implemented by the base R function “cor”. All visualizations were rendered using the “ggplot2” package unless otherwise described. For all analyses, *p* < 0.05 was considered statistically significant, and data are represented as mean ± standard error of mean or mean± standard deviation. Each figure and data set has been provided with individual *P*-values for better clarity.

### Reporting summary

Further information on research design is available in the Nature Research Reporting Summary linked to this article.

## Supplementary information

Supplementary Information

Description of Additional Supplementary Files

Supplementary Data

Reporting Summary

## Data Availability

Microbiome and resistome sequence data that support the findings of this study have been deposited in GenBank with the accession code: PRJNA734321 and PRJNA843121 (https://www.ncbi.nlm.nih.gov/bioproject/PRJNA843121) with the link. All data generated or analyzed during this study are included in this published article (data generated for this study is provided in the Supplementary Data file and public databases-GenBank). Any other data such as certain metagenomic sequences that are not publicly available due to space constraints are available from the corresponding author on reasonable request.

## References

[1] Sabtu N, Enoch DA, Brown NM (2015). Antibiotic resistance: what, why, where, when and how?. Br. Med. Bull..

[2] Bengtsson B, Greko C (2014). Antibiotic resistance-consequences for animal health, welfare, and food production. Ups. J. Med. Sci..

[3] Munita, J. M. & Arias, C. A. Mechanisms of antibiotic resistance. *Microbiol. Spectr*. 10.1128/microbiolspec.VMBF-0016-2015 (2016).

[4] Singh S, Verma N, Taneja N (2019). The human gut resistome: current concepts & future prospects. Indian J. Med. Res..

[5] Wright GD (2010). The antibiotic resistome. Expert Opin. Drug Disco..

[6] Crofts TS, Gasparrini AJ, Dantas G (2017). Next-generation approaches to understand and combat the antibiotic resistome. Nat. Rev. Microbiol.

[7] McInnes RS, McCallum GE, Lamberte LE, van Schaik W (2020). Horizontal transfer of antibiotic resistance genes in the human gut microbiome. Curr. Opin. Microbiol..

[8] Lerminiaux NA, Cameron ADS (2019). Horizontal transfer of antibiotic resistance genes in clinical environments. Can. J. Microbiol..

[9] Partridge, S. R., Kwong, S. M., Firth, N. & Jensen, S. O. Mobile genetic elements associated with antimicrobial resistance. *Clin. Microbiol. Rev.*10.1128/CMR.00088-17 (2018).

[10] Stokes HW, Gillings MR (2011). Gene flow, mobile genetic elements and the recruitment of antibiotic resistance genes into Gram-negative pathogens. FEMS Microbiol. Rev..

[11] Martin D (1992). Operating laparoscopes. Nurs. (Lond.).

[12] Sun J (2020). Environmental remodeling of human gut microbiota and antibiotic resistome in livestock farms. Nat. Commun..

[13] van Schaik W (2015). The human gut resistome. Philos. Trans. R. Soc. Lond. B Biol. Sci..

[14] Phillips ML (2009). Gut reaction: environmental effects on the human microbiota. Environ. Health Perspect..

[15] Ramakrishnan B, Venkateswarlu K, Sethunathan N, Megharaj M (2019). Local applications but global implications: can pesticides drive microorganisms to develop antimicrobial resistance?. Sci. Total Environ..

[16] Bengtsson-Palme, J., Kristiansson, E. & Larsson, D. G. J. Environmental factors influencing the development and spread of antibiotic resistance. *FEMS Microbiol Rev*. 10.1093/femsre/fux053 (2018).

[17] Malagon-Rojas JN, Parra Barrera EL, Lagos L (2020). From environment to clinic: the role of pesticides in antimicrobial resistance. Rev. Panam. Salud Publica.

[18] Nesme J, Simonet P (2015). The soil resistome: a critical review on antibiotic resistance origins, ecology and dissemination potential in telluric bacteria. Environ. Microbiol.

[19] Gao B (2017). Profound perturbation induced by triclosan exposure in mouse gut microbiome: a less resilient microbial community with elevated antibiotic and metal resistomes. BMC Pharm. Toxicol..

[20] Parnanen K (2018). Maternal gut and breast milk microbiota affect infant gut antibiotic resistome and mobile genetic elements. Nat. Commun..

[21] Fitzpatrick MA (2018). Changes in bacterial epidemiology and antibiotic resistance among veterans with spinal cord injury/disorder over the past 9 years. J. Spinal Cord. Med.

[22] Jones BE (2015). Trends in antibiotic use and nosocomial pathogens in hospitalized veterans with Pneumonia at 128 medical centers, 2006–2010. Clin. Infect. Dis..

[23] Kelly AA (2017). A report of the efforts of the veterans health administration national antimicrobial stewardship initiative. Infect. Control Hosp. Epidemiol..

[24] Fitzpatrick MA (2021). Epidemiology and clinical outcomes associated with extensively drug-resistant (XDR) Acinetobacter in US Veterans’ Affairs (VA) medical centers. Infect. Control Hosp. Epidemiol..

[25] Wilson BM (2017). Carbapenem-resistant enterobacter cloacae in Patients from the US veterans health administration, 2006–2015. Emerg. Infect. Dis..

[26] White RF (2016). Recent research on Gulf War illness and other health problems in veterans of the 1991 Gulf War: effects of toxicant exposures during deployment. Cortex.

[27] Janulewicz, P. et al. The multiple hit hypothesis for Gulf War illness: self-reported chemical/biological weapons exposure and mild traumatic brain injury. *Brain Sci*. 10.3390/brainsci8110198 (2018).

[28] Mawson, A. R. & Croft, A. M. Gulf War illness: unifying hypothesis for a continuing health problem. *Int. J. Environ. Res. Public Health*10.3390/ijerph16010111 (2019).

[29] Alhasson F (2017). Altered gut microbiome in a mouse model of Gulf War Illness causes neuroinflammation and intestinal injury via leaky gut and TLR4 activation. PLoS One.

[30] Seth RK (2018). Increased butyrate priming in the gut stalls microbiome associated-gastrointestinal inflammation and hepatic metabolic reprogramming in a mouse model of Gulf War Illness. Toxicol. Appl Pharm..

[31] Janulewicz, P. A. et al. The gut-microbiome in Gulf War veterans: a preliminary report. *Int. J. Environ. Res. Public Health*10.3390/ijerph16193751 (2019).

[32] Saha, P. et al. Andrographolide attenuates gut-brain-axis associated pathology in Gulf War illness by modulating bacteriome-virome associated inflammation and microglia-neuron proinflammatory crosstalk. *Brain Sci.*10.3390/brainsci11070905 (2021).

[33] Toleman MA, Bennett PM, Walsh TR (2006). ISCR elements: novel gene-capturing systems of the 21st century?. Microbiol Mol. Biol. Rev..

[34] Broderick G (2011). A pilot study of immune network remodeling under challenge in Gulf War Illness. Brain Behav. Immun..

[35] Alshelh Z (2020). In-vivo imaging of neuroinflammation in veterans with Gulf War illness. Brain Behav. Immun..

[36] Seth, R. K. et al. Gut DNA Virome diversity and its association with host bacteria regulate inflammatory phenotype and neuronal immunotoxicity in experimental Gulf War illness. *Viruses*10.3390/v11100968 (2019).

[37] Hernandez S, Fried DE, Grubisic V, McClain JL, Gulbransen BD (2019). Gastrointestinal neuroimmune disruption in a mouse model of Gulf War illness. FASEB J..

[38] Kimono D (2020). Host akkermansia muciniphila abundance correlates with Gulf War illness symptom persistence via NLRP3-mediated neuroinflammation and decreased brain-derived neurotrophic factor. Neurosci. Insights.

[39] Butterick TA (2019). Gulf War Illness-associated increases in blood levels of interleukin 6 and C-reactive protein: biomarker evidence of inflammation. BMC Res Notes.

[40] Bose, D. et al. TLR Antagonism by Sparstolonin B alters microbial signature and modulates gastrointestinal and neuronal inflammation in Gulf War illness preclinical model. *Brain Sci*. 10.3390/brainsci10080532 (2020).

[41] Bose, D. et al. Obesity worsens Gulf War illness symptom persistence pathology by linking altered gut microbiome species to long-term gastrointestinal, hepatic, and neuronal inflammation in a mouse model. *Nutrients*10.3390/nu12092764 (2020).

[42] Pawlowska-Kamieniak A, Krawiec P, Pac-Kozuchowska E (2021). Interleukin 6: biological significance and role in inflammatory bowel diseases. Adv. Clin. Exp. Med..

[43] Wright CB (2006). Interleukin-6 is associated with cognitive function: the Northern Manhattan Study. J. Stroke Cerebrovasc. Dis..

[44] Perry JA, Westman EL, Wright GD (2014). The antibiotic resistome: what’s new?. Curr. Opin. Microbiol.

[45] Levy SB, Marshall B (2004). Antibacterial resistance worldwide: causes, challenges and responses. Nat. Med.

[46] Singer AC, Shaw H, Rhodes V, Hart A (2016). Review of antimicrobial resistance in the environment and its relevance to environmental regulators. Front Microbiol.

[47] Gressel J (2011). Low pesticide rates may hasten the evolution of resistance by increasing mutation frequencies. Pest Manag Sci..

[48] Foster KR, Schluter J, Coyte KZ, Rakoff-Nahoum S (2017). The evolution of the host microbiome as an ecosystem on a leash. Nature.

[49] Modak R, Ross D, Kan VL (2008). Macrolide and clindamycin resistance in Staphylococcus aureus isolates and antibiotic use in a Veterans Affairs Medical Center. Infect. Control Hosp. Epidemiol..

[50] Livorsi, D. J. et al. The feasibility of implementing antibiotic restrictions for fluoroquinolones and cephalosporins: a mixed-methods study across 15 Veterans Health Administration hospitals. *J. Antimicrob. Chemother*. 10.1093/jac/dkab138 (2021).

[51] Butler MS, Hansford KA, Blaskovich MA, Halai R, Cooper MA (2014). Glycopeptide antibiotics: back to the future. J. Antibiot. (Tokyo).

[52] Patel N (2021). Comparative effectiveness of early-targeted use of fidaxomicin versus oral vancomycin among hospitalized veterans’ affairs patients with infections due to Clostridioides difficile. Pharmacotherapy.

[53] Caffrey AR, Morrill HJ, Puzniak LA, Laplante KL (2014). Comparative effectiveness of linezolid and vancomycin among a national veterans affairs cohort with methicillin-resistant Staphylococcus aureus pneumonia. Pharmacotherapy.

[54] Gyamlani G (2019). Vancomycin-associated Acute kidney injury in a large veteran population. Am. J. Nephrol..

[55] Tokars JI (1999). The prevalence of colonization with vancomycin-resistant enterococcus at a Veterans’ Affairs institution. Infect. Control Hosp. Epidemiol..

[56] Wang H (2019). Promising treatment for Type 2 diabetes: fecal microbiota transplantation reverses insulin resistance and impaired islets. Front. Cell Infect. Microbiol.

[57] Zhou D (2017). Total fecal microbiota transplantation alleviates high-fat diet-induced steatohepatitis in mice via beneficial regulation of gut microbiota. Sci. Rep..

[58] Craven L (2020). Allogenic fecal microbiota transplantation in patients with nonalcoholic fatty liver disease improves abnormal small intestinal permeability: a randomized control trial. Am. J. Gastroenterol..

[59] Vendrik KEW (2020). Fecal microbiota transplantation in neurological disorders. Front Cell Infect. Microbiol.

[60] Park SH (2021). Cognitive function improvement after fecal microbiota transplantation in Alzheimer’s dementia patient: a case report. Curr. Med. Res. Opin..

[61] Khoruts A, Sadowsky MJ (2016). Understanding the mechanisms of faecal microbiota transplantation. Nat. Rev. Gastroenterol. Hepatol..

[62] Vindigni SM, Surawicz CM (2017). Fecal microbiota transplantation. Gastroenterol. Clin. North Am..

[63] Woodworth MH, Carpentieri C, Sitchenko KL, Kraft CS (2017). Challenges in fecal donor selection and screening for fecal microbiota transplantation: a review. Gut Microbes.

[64] Domingues S, da Silva GJ, Nielsen KM (2012). Integrons: vehicles and pathways for horizontal dissemination in bacteria. Mob. Genet Elem..

[65] Il’ina, T. S. [Mobile ISCR elements: structure, functions, and role in the emergence, increasing and spreading of blocks of bacterial genes of multiple antibiotic resistance]. *Mol. Gen. Mikrobiol. Virusol*. **27**, 3–13 (2012).

[66] Hu Q (2020). Effects of low-dose antibiotics on gut immunity and antibiotic resistomes in weaned piglets. Front. Immunol..

[67] Xia, Y. et al. Combined analysis of metagenomic data revealed consistent changes of gut microbiome structure and function in inflammatory bowel disease. *J. Appl. Microbiol*. 10.1111/jam.15154 (2021).

[68] Keating JA (2019). Characterising the gut microbiome in veterans with Gulf War Illness: a protocol for a longitudinal, prospective cohort study. BMJ Open.

[69] Denkinger CM, Grant AD, Denkinger M, Gautam S, D’Agata EM (2013). Increased multi-drug resistance among the elderly on admission to the hospital-a 12-year surveillance study. Arch. Gerontol. Geriatr..

[70] Giarratano A, Green SE, Nicolau DP (2018). Review of antimicrobial use and considerations in the elderly population. Clin. Inter. Aging.

[71] Augustine S, Bonomo RA (2011). Taking stock of infections and antibiotic resistance in the elderly and long-term care facilities: a survey of existing and upcoming challenges. Eur. J. Microbiol. Immunol. (Bp).

[72] Joshi U (2019). A permethrin metabolite is associated with adaptive immune responses in Gulf War illness. Brain Behav. Immun..

[73] Nkiliza A (2021). Adaptive immune responses associated with the central nervous system pathology of Gulf War illness. Neurosci. Insights.

[74] Lai ZL (2018). Fecal microbiota transplantation confers beneficial metabolic effects of diet and exercise on diet-induced obese mice. Sci. Rep..

[75] Li K (2020). Protection of fecal microbiota transplantation in a mouse model of multiple sclerosis. Mediators Inflamm..

[76] Yan Y, Zhou X, Guo K, Zhou F, Yang H (2020). Chlorogenic acid protects against indomethacin-induced inflammation and mucosa damage by decreasing Bacteroides-derived LPS. Front. Immunol..

[77] Steele, L. et al. Brain-immune interactions as the basis of Gulf War illness: clinical assessment and deployment profile of 1990–1991 Gulf War veterans in the Gulf War Illness Consortium (GWIC) multisite case-control study. *Brain Sci*. **11**, 1132 (2021).

[78] Steele L (2000). Prevalence and patterns of Gulf War illness in Kansas veterans: association of symptoms with characteristics of person, place, and time of military service. Am. J. Epidemiol..

[79] Smets EM, Garssen B, Bonke B, De Haes JC (1995). The Multidimensional Fatigue Inventory (MFI) psychometric qualities of an instrument to assess fatigue. J. Psychosom. Res..

[80] Melzack R (1975). The McGill pain questionnaire: major properties and scoring methods. Pain.

[81] Buysse DJ, Reynolds CF, Monk TH, Berman SR, Kupfer DJ (1989). The Pittsburgh sleep quality index: a new instrument for psychiatric practice and research. Psychiatry Res..

[82] Proctor SP (1998). Health status of Persian Gulf War veterans: self-reported symptoms, environmental exposures and the effect of stress. Int. J. Epidemiol..

[83] (2015).

[84] Beghini, F. et al. Integrating taxonomic, functional, and strain-level profiling of diverse microbial communities with bioBakery 3. *Elife*10.7554/eLife.65088 (2021).

[85] Uritskiy GV, DiRuggiero J, Taylor J (2018). MetaWRAP-a flexible pipeline for genome-resolved metagenomic data analysis. Microbiome.

[86] Nurk S, Meleshko D, Korobeynikov A, Pevzner PA (2017). metaSPAdes: a new versatile metagenomic assembler. Genome Res.

[87] Kang DD (2019). MetaBAT 2: an adaptive binning algorithm for robust and efficient genome reconstruction from metagenome assemblies. PeerJ.

[88] Wu YW, Simmons BA, Singer SW (2016). MaxBin 2.0: an automated binning algorithm to recover genomes from multiple metagenomic datasets. Bioinformatics.

[89] Alneberg J (2014). Binning metagenomic contigs by coverage and composition. Nat. Methods.

[90] Hyatt D (2010). Prodigal: prokaryotic gene recognition and translation initiation site identification. BMC Bioinforma..

[91] Li W, Godzik A (2006). Cd-hit: a fast program for clustering and comparing large sets of protein or nucleotide sequences. Bioinformatics.

[92] Fu L, Niu B, Zhu Z, Wu S, Li W (2012). CD-HIT: accelerated for clustering the next-generation sequencing data. Bioinformatics.

[93] Alcock BP (2020). CARD 2020: antibiotic resistome surveillance with the comprehensive antibiotic resistance database. Nucleic Acids Res..

[94] Potter SC (2018). HMMER web server: 2018 update. Nucleic Acids Res..

[95] Love MI, Huber W, Anders S (2014). Moderated estimation of fold change and dispersion for RNA-seq data with DESeq2. Genome Biol..

