# Supplementary material for: Host gut resistome in Gulf War chronic multisymptom illness correlates with persistent inflammation
Source: Commun Biol. 2022 Jun 7;5:552. doi: 10.1038/s42003-022-03494-7 (PMC9174162; doi:10.1038/s42003-022-03494-7)
Supplement: Supplementary file 1 — Supplementary Information [file 42003_2022_3494_MOESM1_ESM.pdf]

Supplementary Table 1. List of primers for q-RTPCR analysis of mouse and GW veteran ARGs:

a. Mouse Primers:

SL no.	Gene name	ARO Number	Forward	Reverse
1	***vanXYN***	ARO:3002969	TGATGGTCATCGAACGGTTG	CAGCCAGGAGATGCTACATAAC
2	***adeS***	ARO:3000549	CCGAGTTTCGGGACGATTTA	CCCATCTCTTCATGACCCATAG

b. Human Primers:

SL no.	Gene name	ARO Number	Forward	Reverse
1	***mexD***	ARO:3000801	GTCAAGTCCGTGCACAGTATAA	GTTCACACGAGCAACCAATTC
2	***novA***	ARO:3002522	TCACGTTGGACAAAGCGTATC	GGTCATCTGCACCAGCATATAG
3	**Agrobacterium fabrum chloramphenicol acetyltransferase (*afca*)**	ARO:3004451	AACTCGCCGCGTCAATATAA	GGCCAGGTTTCCTGTATCAA

c. 16S Primer:

SL no.	Gene name	Sequence
1	***16S_1237 F***	GGGCTACACACGYGCWAC
2	***16S_1391 R***	GACGGGCGGTGTGTRCA

Supplementary Table 2. Demographic Information of GWI veterans

	Hum_Control	Hum_GWI
N	5	28
Age (years), mean (SD)	55.5 (4.8)	53.9 (5.7)
Height (In), mean (SD)	69.8 (4.6)	68.0 (3.0)
Weight (Lbs), mean (SD)	243.0 (35.3)	214.9 (45.4)
BMI, mean (SD)	35.3 (5.7)	32.7 (6.4)
Average Number of GI symptoms, mean	0.6	2.9
Diarrhea, n (%)	0 (0)	17 (61)
Nausea or Upset Stomach, n (%)	2 (40)	17 (61)
Diarrhea, n (%)	1 (20)	16 (57)
Nausea or Upset Stomach, n (%)	0 (0)	13 (46)
Other Gastrointestinal Disorder, n (%)	0 (0)	15 (54)
