# Supplementary material for: Host gut resistome in Gulf War chronic multisymptom illness correlates with persistent inflammation
Source: Commun Biol. 2022 Jun 7;5:552. doi: 10.1038/s42003-022-03494-7 (PMC9174162; doi:10.1038/s42003-022-03494-7)
Supplement: Supplementary file 4 — Reporting Summary [file 42003_2022_3494_MOESM4_ESM.pdf]

nature portfolio

Corresponding author(s): SAURABH CHATTERJEE

Last updated by author(s): May 5, 2022

Reporting Summary

Nature Portfolio wishes to improve the reproducibility of the work that we publish. This form provides structure for consistency and transparency in reporting. For further information on Nature Portfolio policies, see our Editorial Policies and the Editorial Policy Checklist.

Statistics

For all statistical analyses, confirm that the following items are present in the figure legend, table legend, main text, or Methods section.

n/a	Confirmed	
☐	☒	The exact sample size (n) for each experimental group/condition, given as a discrete number and unit of measurement
☐	☒	A statement on whether measurements were taken from distinct samples or whether the same sample was measured repeatedly
☐	☒	The statistical test(s) used AND whether they are one- or two-sided<br>*Only common tests should be described solely by name; describe more complex techniques in the Methods section.*
☐	☒	A description of all covariates tested
☐	☒	A description of any assumptions or corrections, such as tests of normality and adjustment for multiple comparisons
☐	☒	A full description of the statistical parameters including central tendency (e.g. means) or other basic estimates (e.g. regression coefficient) AND variation (e.g. standard deviation) or associated estimates of uncertainty (e.g. confidence intervals)
☐	☒	For null hypothesis testing, the test statistic (e.g. F, t, r) with confidence intervals, effect sizes, degrees of freedom and P value noted<br>*Give P values as exact values whenever suitable.*
☒	☐	For Bayesian analysis, information on the choice of priors and Markov chain Monte Carlo settings
☒	☐	For hierarchical and complex designs, identification of the appropriate level for tests and full reporting of outcomes
☐	☒	Estimates of effect sizes (e.g. Cohen's d, Pearson's r), indicating how they were calculated

Our web collection on statistics for biologists contains articles on many of the points above.

Software and code

Policy information about availability of computer code

Data collection	Data was collected by whole genome shotgun sequencing performed using next generation sequencing platform by COSMOSID (acknowledged in the manuscript).
Data analysis	MetaPhlAn v3.0.7<br>metaWRAP v1.3.2<br>metaSPAdes v3.13.0<br>metaBAT2 v2.12.1<br>MaxBin2 v2.2.6<br>CONCOCT v1.0.0<br>MetaProdigal v2.6.3<br>HMMER v3.3.1<br>R v3.6.3<br>Graphpad Prism v9<br>All specific R packages are described in the methods section of the study. Workflows describing the use of these tools are described in the methods section of the study, custom pipelines and processing scripts are available from the corresponding author upon reasonable request.

For manuscripts utilizing custom algorithms or software that are central to the research but not yet described in published literature, software must be made available to editors and reviewers. We strongly encourage code deposition in a community repository (e.g. GitHub). See the Nature Portfolio guidelines for submitting code & software for further information.

Data

Policy information about availability of data

All manuscripts must include a data availability statement. This statement should provide the following information, where applicable:

- Accession codes, unique identifiers, or web links for publicly available datasets
- A description of any restrictions on data availability
- For clinical datasets or third party data, please ensure that the statement adheres to our policy

Microbiome and resistome sequence data that support the findings of this study have been deposited in GenBank with the accession code : PRJNA734321 with the link: https://www.ncbi.nlm.nih.gov/sra/PRJNA734321 (also mentioned in the manuscript).

Field-specific reporting

Please select the one below that is the best fit for your research. If you are not sure, read the appropriate sections before making your selection.

☒ Life sciences ☐ Behavioural & social sciences ☐ Ecological, evolutionary & environmental sciences

For a reference copy of the document with all sections, see nature.com/documents/nr-reporting-summary-flat.pdf

Life sciences study design

All studies must disclose on these points even when the disclosure is negative.

Sample size	For preclinical GWI persistence mouse model, the sample size was chosen based established mouse models of GWI where n=11 mice in the Control group, n=11 mice in the GWI group, and n=6 mice in the GWI_FMT group were used. In GWI Veteran groups, we had 5 samples in the Hum_Control group and 28 samples in the Hum_GWI group.
Data exclusions	No data were excluded in the analysis except for very low read values in the antibiotic resistance genes (ARG) and mobile genetic elements (MGE) analysis of mouse and Veteran samples.
Replication	For qRT-PCR, each mouse and human samples were run in triplicates. For ELISA, mouse and human serum samples were run in duplicates. Immunohistochemistry was performed with all the samples from 3 mice groups and images were taken from 2 different fields in each sample (only one representative image of each group is shown).
Randomization	For preclinical GWI persistence mouse model, randomization was performed. Initially, 18 adult, male, C57BL/6J mice of age 10 weeks were randomly divided into 2 groups of n=6 and n=12. In a parallel experiment, 10 additional adult, male, C57BL/6J mice of same age were randomly divided into the same two groups (n=5 mice/ group) and added to the existing Control and GWI groups. Finally, the mice group (n=11) was considered as Control group and vehicle (0.6% DMSO) was administered. The mice group (n=17) was considered GWI and administered with GWI chemical Pyridostigmine bromide(2mg/kg) and Permethrin(200mg/kg) triweekly for 15 days. After 15 days of GWI chemical dosing, 6 mice were randomly chosen to receive FMT for next 20 weeks and formed the GWI_FMT group. The remaining 11 mice formed the GWI group.
Blinding	No blinding was performed during sequencing of DNA from mouse and human fecal sample as it is not possible for investigators to influence the process.

Reporting for specific materials, systems and methods

We require information from authors about some types of materials, experimental systems and methods used in many studies. Here, indicate whether each material, system or method listed is relevant to your study. If you are not sure if a list item applies to your research, read the appropriate section before selecting a response.

Materials & experimental systems

n/a	Involved in the study	
☐	☒	Antibodies
☒	☐	Eukaryotic cell lines
☒	☐	Palaeontology and archaeology
☐	☒	Animals and other organisms
☐	☒	Human research participants
☒	☐	Clinical data
☒	☐	Dual use research of concern

Methods

n/a	Involved in the study	
☒	☐	ChIP-seq
☒	☐	Flow cytometry
☒	☐	MRI-based neuroimaging

Antibodies

Antibodies used	Primary IL-1b antibody (catlog no.: sc-52012, lot no.: G0119) was purchased Santacruz Biotechnology (Dallas, TX, USA).<br>Primary BDNF antibody (catlog no.: sc-65514, lot no.: K1519) was purchased Santacruz Biotechnology (Dallas, TX, USA).<br>Secondary anti-mouse biotinylated antibody (catlog no.: BA-9200, lot no.: ZF0909) was purchased Vector laboratories (Burligame,CA,USA).
Validation	Validation was done by the manufacturers and the validation report of the respective manufacturers will be provided by the corresponding author upon reasonable request.

Animals and other organisms

Policy information about studies involving animals; ARRIVE guidelines recommended for reporting animal research

Laboratory animals	Adult, C57BL/6J, male mice of 10 weeks age were purchased from Jackson Laboratories (Bar Harbor, ME, USA).
Wild animals	N/A
Field-collected samples	N/A
Ethics oversight	All animal experimental procedures were approved by the IACUC (approved protocol number 2419-101345-072318) on 07/23/2020, University of South Carolina at Columbia, SC.

Note that full information on the approval of the study protocol must also be provided in the manuscript.

Human research participants

Policy information about studies involving human research participants

Population characteristics	The Boston Gulf War Illness Consortium (GWIC) included Veterans deployed in the Gulf War i.e. from August 1990 to July 1991. The GWIC used the Kansas GWI criteria and the Veterans who met the required criteria were assigned as GWI group. Those Veterans who did not meet Kansas criteria are deemed the Control group.
Recruitment	The GWIC recruited the GWI veterans as participants via telephone on completion of GWIC study protocol. After filling out a brief questionnaire regarding screening, the participants were sent a stool collection kit which was then shipped back to the investigators (details mentioned in the manuscript).
Ethics oversight	The protocol was approved by Institutional Review Board at Boston University School of Public Health (proposal no. GW170068) on 4/15/2021.

Note that full information on the approval of the study protocol must also be provided in the manuscript.
